# Creation of de novo cryptic splicing for ALS/FTD precision medicine

**DOI:** 10.1126/science.adk2539

**Published:** 2024-10-03

**Authors:** Oscar G. Wilkins, Max Z.Y.J. Chien, Josette J. Wlaschin, Simone Barattucci, Peter Harley, Francesca Mattedi, Puja R. Mehta, Maria Pisliakova, Eugeni Ryadnov, Matthew J. Keuss, David Thompson, Holly Digby, Lea Knez, Rebecca L. Simkin, Juan Antinao Diaz, Matteo Zanovello, Anna-Leigh Brown, Annalucia Darbey, Rajvinder Karda, Elizabeth M.C. Fisher, Thomas J. Cunningham, Claire E. Le Pichon, Jernej Ule, Pietro Fratta

**Affiliations:** 1 UCL Queen Square Motor Neuron Disease Centre, Department of Neuromuscular Diseases, UCL Queen Square Institute of Neurology, UCL; London, WC1N 3BG, UK; 2 The Francis Crick Institute; London, NW1 1AT, UK; 3 *Eunice Kennedy Shriver* National Institute of Child Health and Human Development, National Institutes of Health; Bethesda, MD 20892, USA; 4 Department of Biology, Johns Hopkins University, Baltimore, MD 21218, USA; 5 Mammalian Genetics Unit, MRC Harwell Institute; Oxfordshire, OX11 0RD, UK; 5a MRC Prion Unit at UCL and UCL Institute of Prion Diseases, London, W1W 7FF, UK; 6 UK Dementia Research Institute at King’s College London, London, SE5 9RX, UK; 7 EGA-Institute for Women’s Health, University College London; London, WC1E 6HX, UK

## Abstract

Loss-of-function of the RNA-binding protein TDP-43 (TDP-LOF) is a hallmark of amyotrophic lateral sclerosis (ALS) and other neurodegenerative disorders. Here we describe TDP-REG, which exploits the specificity of cryptic splicing induced by TDP-LOF to drive protein expression when and where the disease process occurs. The SpliceNouveau algorithm combines deep learning with rational design to generate customizable cryptic splicing events within protein-coding sequences. We demonstrate that expression of TDP-REG reporters is tightly coupled to TDP-LOF *in vitro* and *in vivo*. TDP-REG enables genomic prime-editing to ablate the UNC13A cryptic donor splice site specifically upon TDP-LOF. Finally, we design TDP-REG vectors encoding a TDP-43/Raver1 fusion protein which rescues key pathological cryptic splicing events, paving the way for the development of precision therapies for TDP43-related disorders.

Amyotrophic lateral sclerosis (ALS) is a devastating and incurable neurodegenerative disease. In 97% of cases, there is pronounced formation of neuronal cytoplasmic aggregates of the RNA-binding protein TDP-43 (1). Further, such pathology is found in ~45% of frontotemporal dementia (FTD) cases, and is also observed in limbic-predominant age-related TDP-43 encephalopathy (LATE) and Alzheimer’s disease, suggesting TDP-43-mediated RNA dysregulation is a critical element in many neurodegenerative diseases (2, 3).

TDP-43 is a key regulator of splicing, protecting the transcriptome from toxic “cryptic exons” (CEs), which become prominently expressed upon TDP-43 loss-of-function (TDP-LOF); CEs typically introduce premature termination codons (PTCs) into transcripts, preventing the expression of crucial proteins including STMN2 and UNC13A (4–9). We and others previously showed that genetic modulation of even a single cryptic exon can influence disease progression, meaning that therapeutics active in the stage in which cryptic exons are expressed could still impact disease course (7, 8). Numerous preclinical studies aim to reduce TDP-43-associated toxicity, for example via blocking cryptic exon inclusion using antisense oligonucleotides (ASOs) or transgenes, or by targeting the aggregation process itself (4, 10–12).

A major barrier to bringing gene therapy approaches to the clinic is the lack of methods to tightly regulate transgene expression. Only a small fraction of motor neurons, let alone all neurons, display clear pathology at any given time in patients (13). Therefore, a conventional targeting approach (for example, combining an AAV serotype with CNS-tropism with a neuronal promoter) would result in expression of the therapeutic transgene in a vast number of non-degenerating cells. Such expression is not merely unnecessary, but could worsen prognosis by interfering with the homeostasis of otherwise healthy cells, especially given the permanent nature of these approaches (14). Such concerns recently led to the development of therapeutics with activity-dependent transcriptional promoters for epilepsy (15).

Here, we leverage our understanding of TDP-43’s regulation of splicing together with deep-learning-based splicing prediction tools to generate vectors featuring novel cryptic splice sites. Crucially, whereas pathological cryptic splicing blocks protein expression, our vectors explicitly require the use of the cryptic splice site(s) to express the encoded transgene. This approach limits expression of transgenes to cells with TDP-LOF, which co-occurs with TDP-43 aggregation in patients, thus reducing the risk of off-target side effects. In addition to demonstrating highly effective TDP-LOF-dependent expression *in vitro* and *in vivo*, we apply this approach to two candidate ALS/FTD gene therapies, paving the way toward safer and more efficacious treatments for these devastating diseases.

## Modification of ALS/FTD cryptic exons for TDP-43-regulated expression vectors

**Fig. 1:**
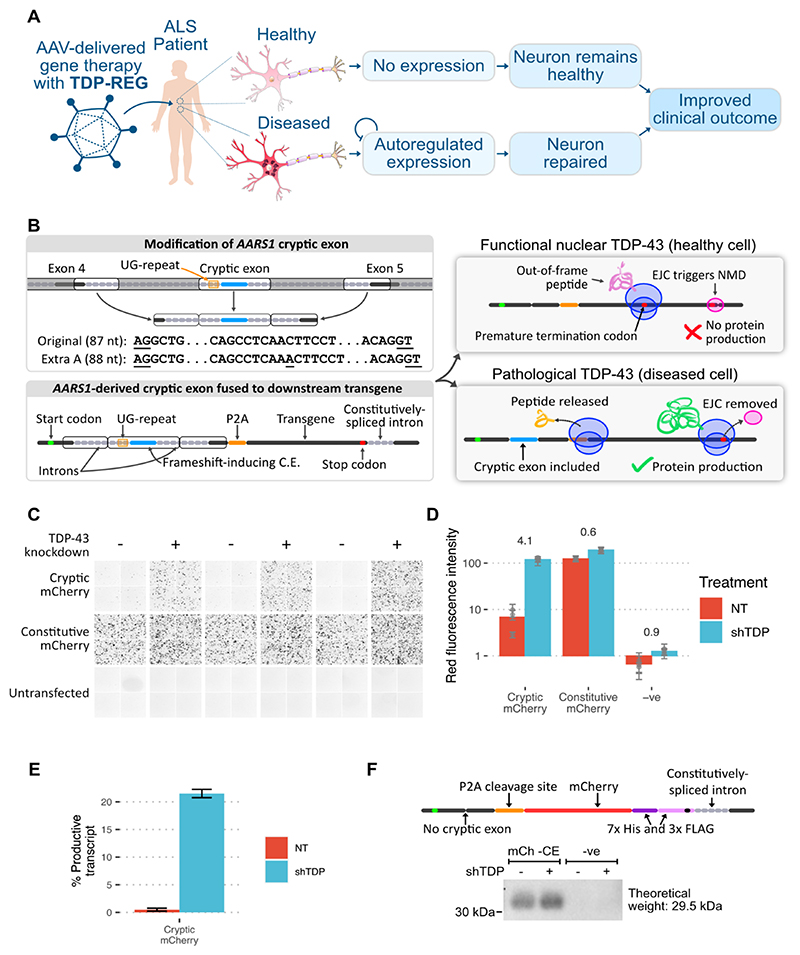
Regulatory upstream CE controls downstream transgene expression (TDP-REGv1). **A:** Schematic showing the intended function and purpose of this work. **B:** Schematic of TDP-REGv1. *Top left:* the genomic locus surrounding the human *AARS1* CE region was modified, with the middle parts of the introns removed to reduce size, and an extra adenosine added to the CE sequence to enable frame-shifting. The AG and GT splice sites of the CE are underlined. *Bottom left:* the modified sequence from part A was incorporated into a “minigene”. *Top right*: when the CE is excluded, the start codon is out-of-frame with the transgene, triggering nonsense-mediated decay (NMD) due to the downstream exon junction complex (EJC). *Bottom right:* when the CE is included, the transgene is in-frame with the start codon, resulting in protein expression. **C**: Fluorescence microscopy images (red channel) showing SK-N-BE(2) cells with (shTDP) or without (NT = “not treated”) TDP-43 knockdown, transfected with a vector fusing the upstream *AARS1-*derived sequence to mCherry (“cryptic mCherry”) or a constitutive vector. **D**: Quantification of the images in Part C; numbers show log2-fold-change in TDP-43 knockdown cells; each dot shows the average of one well (three wells per condition), with error bars showing the standard deviation within each well (four per well). **E**: Summary of Nanopore sequencing results for the cells in Part C; error bars show standard deviation across three replicates. **F**: Top: schematic of mCh -CE vector, without the CE but with a downstream His/Tri-FLAG dual tag to enable sensitive detection. Bottom: Western blot of cells transduced with the above vector (“mCh -CE”) or a completely different vector (“-ve” - a Prime Editing vector). Samples were enriched with a His-tag pulldown, then blotted with an anti-FLAG antibody.

Numerous TDP-43-regulated CEs have been reproducibly detected in cellular and animal models, showing high specificity for TDP-LOF; furthermore, a number of these, including CEs in *STMN2, UNC13A, HDGFL2* and *AARS1*, have been found specifically within neurons with TDP-43 pathology in post-mortem ALS and FTD samples (4–8, 16). We reasoned that a cryptic exon with these features could be used to regulate expression of a therapeutic or reporter transgene (Fig. 1A). We identified the cryptic exon in the *AARS1* gene (hg38: chr16:70272796-70272882) as the most suitable candidate due to its reproducible detection, its lack of stop codons in at least one frame, and its short, defined TDP-43 binding motif (Fig. 1B; fig. S1). We added an extra residue to the cryptic exon to cause frame-shifting, and removed sections of the flanking introns that were likely unnecessary for regulation by TDP-43 (Fig. 1B; see Methods). We then placed this sequence between an upstream start codon and downstream transgene such that only when the cryptic exon was included would the start codon be in-frame with the transgene (Fig. 1B). We included a P2A “self-cleavage” site so that the upstream peptide encoded by the CE is released separately from the main protein (Fig. 1B). Furthermore, we added a constitutively-spliced intron derived from *RPS24* to promote nonsense-mediated decay (NMD) if a PTC is encountered. We refer to this type of construct as TDP-REGv1.

We used mCherry as the transgene to visualize whether this system restricted expression to cells with TDP-LOF. This reporter construct, along with a positive control in which the cryptic exon sequence is constitutively expressed, was transfected into SK-N-BE(2) cells (a human neuroblastoma cell line) with doxycycline-inducible TDP-43 knockdown. A >16-fold increase in mCherry expression was detected in cells with TDP-43 knockdown when using the cryptic construct (Fig. 1C-D). In agreement with this, Nanopore sequencing revealed a >43-fold increase in cryptic exon inclusion upon TDP-43 knockdown (Fig. 1E). We noted that in cells with normal TDP-43, mild leaky expression of mCherry protein was detectable, even though cryptic exon inclusion was less than 0.5%, suggesting a mechanism of protein translation that circumvents the presence of the upstream frameshift. Using western blotting, we observed low-degree leaky expression even in a vector in which the cryptic exon is deleted, potentially due to use of alternative transcriptional start sites or leaky ribosomal scanning (Fig. 1F).

## Creation of de novo cryptic splicing sequences

**Fig. 2:**
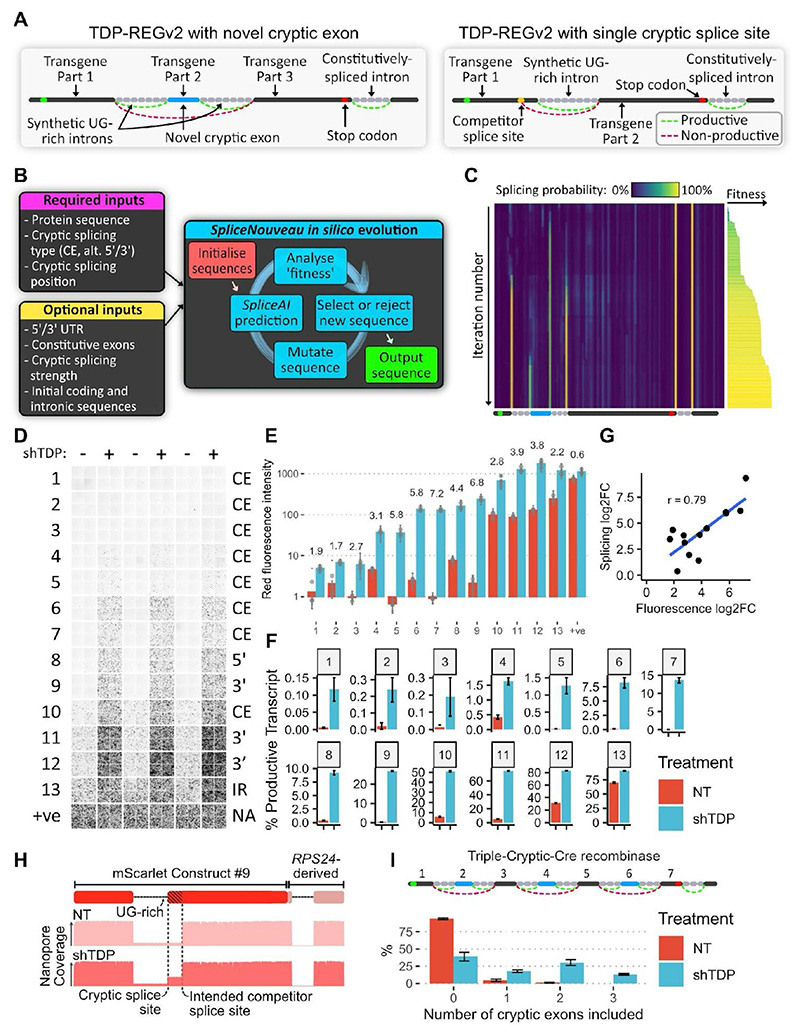
Deep-learning-guided design of novel cryptic splicing events (TDP-REGv2): **A:** Diagrams of internal cryptic exon (left) and single intron (right) designs; for the single intron design, an alternative 5’ splicing design is shown. **B:** Schematic of the *SpliceNouveau* algorithm for designing new cryptic splicing-dependent expression vectors. **C:** Heatmap showing the *in silico* evolution trajectory for an example internal cryptic exon design (below), with associated “fitness” of each (right), which was initialized with a constitutively spliced intron from *RPS24* at the 3’ end. As the iteration number increases, splice sites are “evolved” at the desired positions, and off-target splice sites are depleted. The splice sites flanking the cryptic exon are weaker than the constitutive splice sites, as specified by the user. **D:** Fluorescence microscopy images showing mScarlet expression for 13 constructs generated by *SpliceNouveau* and a positive control. Each replicate consists of four images taken from different parts of the well. **E:** Quantification of D; each dot represents the average of the four images for each replicate; error bars show the standard deviation of images in each well. **F:** Percentage of productive transcripts (transcripts which are predicted to produce full-length mScarlet), as determined by Nanopore sequencing; error bars show standard deviation across three replicates. **G:** Correlation of fold changes in fluorescence and productive isoform fraction; Pearson correlation shown. **H**: Diagram of mScarlet construct 9 (top); representative Nanopore pileup with and without TDP-43 knockdown (bottom). **I:** Schematic of construct encoding Cre recombinase, split across seven exons; exons 2, 4 and 6 are flanked by UG-rich regions (top); number of cryptic exons included in each transcript without and with shTDP, assessed by Nanopore sequencing (bottom); error bars show standard deviation across three replicates.

As a next step, we reasoned that if new CEs could be created within the transgene-encoding region of the vector, this would remove any risk of leaky expression when the CE is spliced out because the full, uninterrupted transgene coding sequence would not be present in the mature mRNA. Further, this approach would avoid expression of unwanted upstream peptides and reduce the vector size. In pilot tests, we found that the *AARS1-*derived cryptic exon could be replaced with other sequences, and that the cryptic exon strength of these sequences correlated with SpliceAI splicing predictions (fig. S2A-D) (17). Encouraged by this, we built an *in silico* directed evolution algorithm, *SpliceNouveau*, which combines rational design principles with SpliceAI predictions, to design *de novo* TDP-43-regulated cryptic cassette exons or single cryptic splice sites (Fig. 2A-C). TDP-43 preferentially binds to stretches of UG-repeats (18, 19).

We therefore programmed *SpliceNouveau* to incorporate different types of UG-rich regions into our designs, including upstream UG-repeats (as in *AARS1*), downstream UG-repeats (as in *Sars*), upstream and downstream repeats (as in *Smg5*) or UG-rich regions without extended UG-repeats (as in *UNC13A*) (fig. S3A).

To create single-intron vectors, which can be smaller than those based on CEs flanked by two introns, we modified our algorithm to design candidate alternative 5’ or alternative 3’ splice sites, which compete with the designed cryptic splice site (Fig. 2A). In each case, the single intron was heavily enriched for TDP-43 binding sites; for alternative 3’ splice sites, the algorithm optimized part of the coding sequence to produce a polypyrimidine-rich region. Using *SpliceNouveau*, we also attempted to design variants exhibiting TDP-43-regulated intron retention by specifying weaker splice sites with lower SpliceAI scores combined with a single UG-rich intron. We refer to constructs generated using this approach as TDP-REGv2.

Of the twenty-seven designs that were tested in SK-N-BE(2) cells, thirteen resulted in increased expression upon TDP-43 knockdown (Fig. 2D-H). Many constructs achieved greater dynamic range than our initial *AARS1*-based design, with two constructs achieving >100-fold increases in expression. The maximal expression varied greatly across constructs, from >100-fold less to greater than the positive control, thus demonstrating that expression can be fine-tuned (Fig. 2E). Via targeted Nanopore sequencing of 56 vectors, we confirmed that those with higher optimization were more likely to be spliced in the expected manner (fig. S4). Overall, only a modest number of vectors need to be experimentally screened to identify those with the desired splicing properties.

Seven of the successful designs were further tested in an SH-SY5Y cell line (human neuroblastoma cell line) with inducible TDP-43 knockdown (7), which yielded very similar results (fig. S3B-C). To demonstrate that the splicing regulation was due specifically to TDP-43 depletion, we co-transfected a selection of the above constructs into SK-N-BE(2) cells with TDP-43 knockdown, together with either functional TDP-43/Raver1 fusion protein, which has been shown to rescue TDP-LOF, or an inactive TDP-43/Raver1 fusion protein with impaired binding to RNA (“2FL”) (4, 18). Cells co-transfected with the inactive 2FL mutant exhibited greatly increased fluorescence compared to cells co-transfected with functional TDP-43/Raver1 (fig. S5A). Furthermore, whereas shRNAs against TDP-43 dramatically increased fluorescence, shRNAs against other key splicing regulators did not (fig. S5B-C).

## Multiple cryptic exons further increase specificity

Next, we examined whether multiple cryptic exons could be included within the same construct, further reducing the risk of leaky expression. We designed a construct encoding Cre recombinase enzyme split across seven exons, three of which were cryptic (Fig. 2I). Nanopore sequencing revealed that in untreated cells <0.05% of transcripts featured all three cryptic exons, whereas shTDP cells expressed all three cryptic exons in approximately 10% of transcripts (Fig. 2I).

Furthermore, lower leaky expression was detected for the construct with three CEs versus constructs with only one or two CEs (fig. S6).

## TDP-REG is activated by TDP-LOF *in vivo*

We then examined whether TDP-REG constructs are also functional *in vivo* in mammalian spinal cord, the main target of ALS therapeutic approaches. We injected AAVs (PHP.eB serotype with hSynapsin promoter) containing TDP-REGv1 mCherry or TDP-REGv2 mScarlet (construct #7) into TDP-43 conditional knockout (cKO) mice (TDP-43^Fl/Fl^;Chat-Cre^+/wt^), where TDP-43 loss was driven by a ChAT-Cre driver line and therefore directed, within the spinal cord, to motor neurons and other spinal cholinergic cells.

**Fig. 3:**
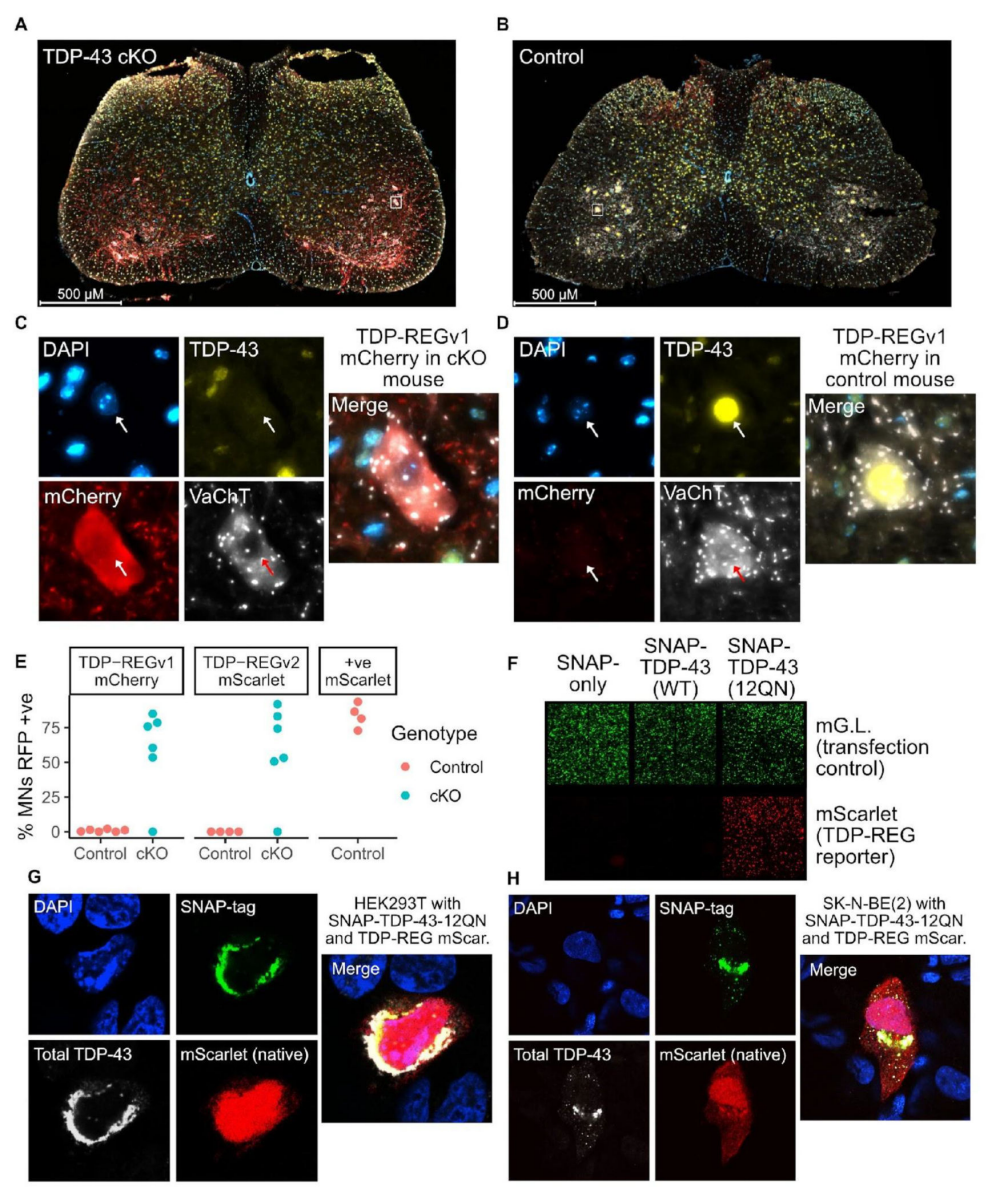
Functionality of TDP-REG in different biological contexts: **A:** Spinal cord of a TDP-43 cKO mouse injected with TDP-REGv1 mCherry AAV (blue = DAPI, yellow = TDP-43, red = mCherry, white = VAChT). **B:** Equivalent to part A, but with a control mouse. **C** and **D:** Magnified representative motor neurons from figures in parts **A** and **B** respectively; white boxes show regions of magnification. **E:** Quantification of the percentage of motor neurons (MNs) with clear mCherry (TDP-REGv1 mCherry), mScarlet (TDP-REGv2 mScarlet #7 - see fig. S7A) or positive control mScarlet for cKO and control mice (only control mice for positive control AAV; see fig S8B); N = 4-6 per condition. **F:** Representative fluorescence microscopy images of HEK293T cells transduced with TDP-REGv2:mScarlet #7, a constitutive mGreenLantern vector, and expression vectors for SNAP-tag only, SNAP-tag-TDP-43(Wild-type) or SNAP-tag-TDP-43(12QN mutant); see also fig. S7C for N=6 replicates. **G** and **H**: Confocal microscopy of HEK293T and SK-N-BE(2) cells, respectively, co-transfected with TDP-REGv2:mScarlet reporter (#7) and SNAP-TDP-43-12QN.

We observed striking expression of mCherry or mScarlet in the motor neurons of these mice, with at least 50% of identified motor neurons having clear expression in all but one case for each construct (Fig. 3A, 3C, 3E; fig. S7A). By contrast, control mice (TDP-43^Fl/wt^;Chat-Cre^+/wt^) displayed very little expression of mCherry, with between 0% and 2% of identified motor neurons having detectable mCherry or mScarlet expression (Fig. 3B, 3D, 3E; fig. S7A). A positive control mScarlet AAV without TDP-REG showed no such specificity when injected into control mice, instead strongly expressing mScarlet in the majority of TDP-43-positive motor neurons (Fig. 3E, fig. S7B).

## TDP-43 aggregation activates TDP-REG expression

We then tested whether TDP-REG vectors could be activated by TDP-43 cytoplasmic aggregation, which would more accurately simulate the disease process than TDP-43 knockdown. We co-transfected TDP-REGv2:mScarlet #7 (and an mGreenLantern transfection control) into HEK293T cells with an aggregation-prone version of TDP-43 in which the Q/N-rich domain is repeated twelve times (SNAP-TDP-43-12QN) (20). We observed strong expression of mScarlet induced by SNAP-TDP-43-12QN but not by SNAP-TDP-43 (wild-type) or SNAP-tag alone (Fig. 3F; fig S7C). Using confocal microscopy, we confirmed that, in a subset of cells, SNAP-TDP-43-12QN was highly expressed and heavily enriched in the cytoplasm, activating TDP-REG mScarlet (construct #7) expression (Fig. 3G-H). Therefore, TDP-REG is functional *in vivo* and is activated by TDP-43 aggregation, strongly suggesting this approach will function within ALS/FTD patients.

## Application of TDP-REG to biomarker expression

Although TDP-LOF is an established key feature of ALS progression, there is a lack of tools to detect it in cell and animal models of disease, thus limiting preclinical studies. In addition to the fluorescent reporters described above, we generated a sensitive luminescent biomarker that could potentially be adapted to monitor TDP-LOF in organoid and animal models. We fused the Gaussia Princeps luciferase (Gluc) sequence downstream of the *AARS1-*based minigene described above (TDP-REGv1), and also used *SpliceNouveau* to create five vectors encoding Gluc with an internal, synthetic cryptic exon (TDP-REGv2). All of these vectors featured increased expression of productively-spliced transcripts upon TDP-43 knockdown (fig. S8A).

**Fig. 4:**
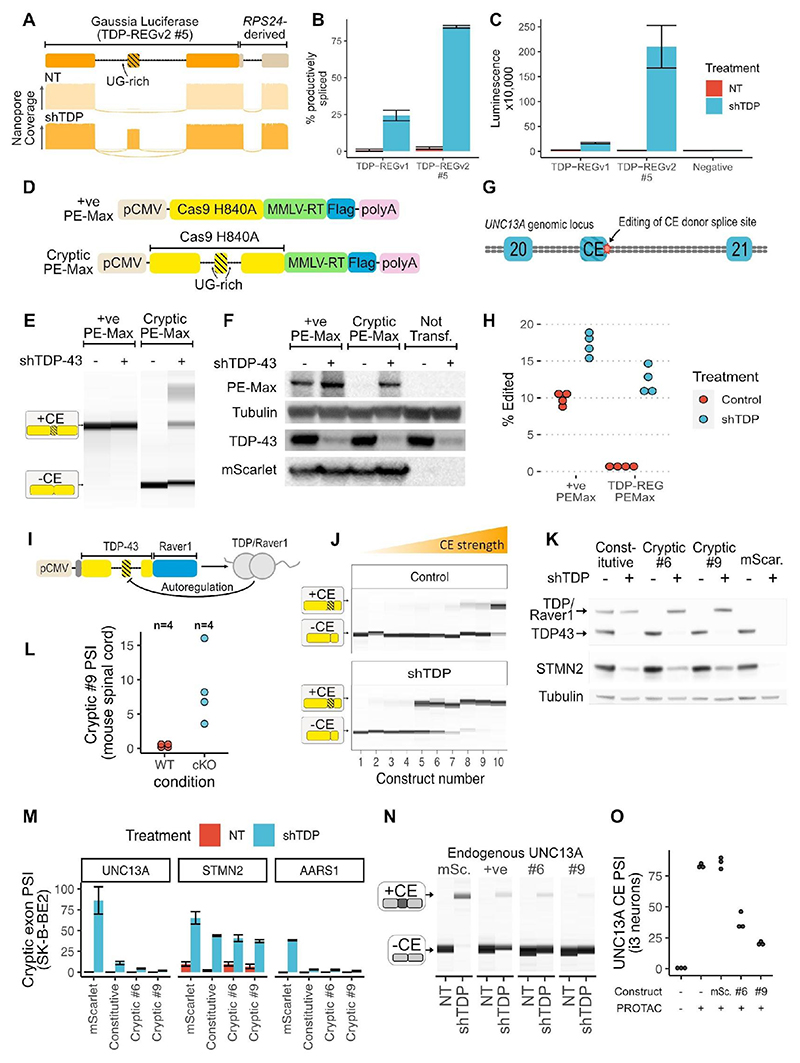
Biosensors, genome editing and splicing rescue: **A:** Schematic of TDP-REGv2 Gluc vector #5 (top); representative Nanopore pileups with (shTDP) and without (NT = “not treated” with doxycycline) TDP-43 knockdown (bottom). **B**: Quantification of Nanopore data for Gluc TDP-REGv1 and TDP-REGv2 #5 for SK-N-BE(2) cells with and without TDP-43 knockdown; error bars show standard deviation across four replicates. **C:** Quantification of luminescence from supernatant of cells used for Part B; error bars show standard deviation across four replicates. **D**: Schematic of constitutive and cryptic (TDP-REGv2) Prime Editing vector (based on Addgene PE-Max vector). **E**: Capillary electrophoresis results from RT-PCR of cryptic or constitutive PE-Max vector transfected into SK-N-BE(2) cells with or without TDP-43 knockdown. **F:** Western blot of FLAG-tagged PE-Max vectors (co-transfected with FLAG-tagged mScarlet) in SK-N-BE(2) cells with or without TDP-43 knockdown; representative blot of N=3 blots. **G:** Diagram of *UNC13A* genomic locus surrounding the *UNC13A* CE between exons 20 and 21; the position of genome editing is shown. **H**: Quantification of intended editing of *UNC13A* in SK-N-BE(2) cells with either vector, +/- TDP-43 knockdown, assessed by targeted Nanopore sequencing. **I:** Schematic of TDP-43/Raver1 with an internal cryptic exon. The design features an additional dual N-terminal NLS; the constitutive vector encodes the same amino acid sequence, but with the introns removed. **J:** RT-PCR analysis of ten constructs designed (as in part A), using the 2FL mutation to block auto-repression of the cryptic exon; constructs were transfected into SK-N-BE(2) cells without (NT) or with (shTDP) doxycycline-induced TDP-43 knockdown; representative traces from N=3 traces. **K:** Western blot of SK-N-BE(2) cells stably expressing constitutive or cryptic (vectors 6 and 9) TDP-43/Raver1, or mScarlet, with or without endogenous TDP-43 knockdown. Endogenous TDP-43 and TDP-43/Raver1 are labeled in the anti-TDP-43 blot; STMN2 expression is visible in the middle blot; tubulin loading control is shown below. All lines are polyclonal piggyBac lines; three polyclonal lines were made per construct with consistent results; representative blot of N=3 blots. **L:** Nanopore-based quantification of splicing of vector #9 when expressed in the spinal cord (via AAV injection) of wild-type or TDP-43 cKO mice. **M:** Quantification of UNC13A, STMN2, and AARS1 cryptic splicing, using all three polyclonal replicates; error bars show standard deviation across three replicates (one replicate for mScarlet control “NT” was excluded because the RNA failed QC). **N:** RT-PCR analysis of endogenous UNC13A cryptic splicing for the same lines as Part K, showing results from a single replicate. **O:** Quantification of RT-PCRs against UNC13A CE in polyclonal Cortical i3 neuron lines, derived from a clonal line in which TDP-43 is fused to HaloTag enabling PROTAC-mediated inducible degradation, expressing mScarlet or TDP/Raver1 vectors #6 or #9.

Both the TDP-REGv1 vector and the best TDP-REGv2 vectors featured high CE inclusion upon TDP-43 knockdown with minimal leakiness, but the best-performing TDP-REGv2 vector had better dynamic range (Fig. 4A-B): it led to >200-fold increase in Gluc expression upon TDP-43 knockdown in SK-N-BE(2) cells, with negligible leaky expression (Fig. 4C). These vectors could enable sensitive detection of TDP-LOF in disease models and high-throughput screens.

## TDP-REG tightly regulates genome editing

We explored the possibility of performing genome editing to remove cryptic splice sites. The Prime Editing (PE) approach, which avoids the need for highly mutagenic double-stranded breaks, can be adapted for high-efficiency *in vivo* editing of the central nervous system (CNS) (21, 22). However, even PE can lead to unwanted editing events that could potentially be toxic (23). Using *SpliceNouveau*, we designed a TDP-REGv2 vector encoding an optimized PE construct (“PE-Max”) where part of the Cas9 sequence was encoded by a TDP-REG CE (Fig. 4D) (24). Via RT-PCR and western blotting, we confirmed that the CE had near-undetectable leaky expression (Fig. 4E-F).

We then designed a prime editing guide RNA (pegRNA) to edit the donor splice site of the *UNC13A* cryptic exon (Fig. 4G) (7, 8), combined with a nicking sgRNA to improve editing efficiency (21). We tested editing efficiency with or without TDP-43 knockdown in SK-N-BE(2) cells. The constitutive PE-Max vector led to editing regardless of TDP-43 knockdown, whereas the CE-containing vector tightly restricted editing to cells with TDP-43 knockdown (Fig. 4H).

Thus, TDP-REG can ensure that genome editing occurs only in cells where it is beneficial, minimizing the potential for off-target effects when delivered to all cells.

## TDP-REG can enable autoregulated splicing rescue

Finally, we used our system to directly rescue TDP-LOF. Fusions of TDP-43 to a less aggregation-prone splicing repressor domain (such as from *RAVER1*) can partially rescue TDP-43 splicing function (4). However, even small imbalances in TDP-43 expression can cause substantial toxicity, and therefore despite its lesser aggregation, generalized expression of TDP-43/Raver1 fusions is undesirable (25). We used *SpliceNouveau* to design TDP-REGv2 constructs encoding TDP-43 fused to the *Raver1* splicing repressor domain (*TDP-REGv2:Raver1*) featuring an internal CE (Fig. 4I). To ensure that expression of the synthetic CE occurred upon even a mild decrease in TDP-43 function, thus enabling rescue at the first signs of TDP-43 pathology, we specified high SpliceAI scores (60-100%) for the CE splice sites and used short UG-repeats to limit the binding affinity of the introns to TDP-43.

TDP-43/Raver1 fusion protein mimics TDP-43’s splicing repressor function and would therefore be expected to autoregulate by inhibiting expression of the synthetic CEs in these constructs.

Whilst this is a great advantage therapeutically, as it would prevent overexpression of TDP-43/Raver1, it could also mask the responsiveness of our synthetic CEs to TDP-LOF during these initial tests. We therefore used an inactive 2FL mutant during initial RT-PCR screens (18). All but one construct displayed detectable CE expression upon TDP-43 knockdown, with six showing high expression; presumably due to the use of high SpliceAI scores and relatively short UG-rich regions, some vectors had detectable leaky CE expression (Fig. 4J).

Using the piggyBac system (see Methods), we generated polyclonal SK-N-BE(2) lines expressing TDP-43/Raver1 fusion in triplicate, either as a constitutive version or with an internal CE from constructs #6 or #9, or mScarlet. In this case, the wild-type TDP-43 sequence was used to enable both repression of cryptic splicing and autoregulation. Whereas the constitutive vector expressed TDP-43/Raver1 regardless of endogenous TDP-43 amounts, vectors featuring an internal CE only expressed detectable TDP-43/Raver1 upon knockdown of endogenous TDP-43 (Fig. 4K; fig. S8B). We then generated an AAV featuring the most promising vector (#9) and, using Nanopore sequencing, assessed its behavior in the spinal cord of wild-type and TDP-43 cKO mice. We observed a substantial increase in expression of the TDP-43-encoding CE in cKO mice compared with WT mice (Fig. 4L).

Next, we assessed whether these vectors rescue key human cryptic splicing events. *TDP-REGv2:Raver1* vectors were able to repress the *UNC13A* and *AARS1* CEs by over 85%, and the *STMN2* CE by approximately 40% (Fig. 4M-N). Furthermore, whereas knockdown of TDP-43 in cells expressing mScarlet featured essentially undetectable STMN2 protein, cells with TDP-43/Raver1 vectors displayed a rescue consistent with that observed for splicing (Fig. 4K, fig. S8C).

The *UNC13A* CE leads to the loss of a crucial pre-synaptic protein. We therefore assessed its rescue by these constructs in iPSC-derived cortical neurons, which form synapses. *TDP-REGv2:Raver1* vectors were able to rescue UNC13A cryptic splicing in iPSC-derived neurons and also rescued *UNC13A* protein at the synapses (Fig. 4O, fig. S9A-C).

Higher levels of TDP-43/Raver1 expression, and more efficient repression of endogenous CEs, were observed upon TDP-43 knockdown for cryptic vectors (especially #9) than for the constitutive vector (Fig. 4K, 4M-N). We propose this is due to toxicity of TDP-43/Raver1 when expressed in cells with functional endogenous TDP-43, leading to a selection pressure against cells with high constitutive expression of TDP-43/Raver1. Indeed, a strong growth advantage was observed for cells expressing TDP-43/Raver1 regulated by TDP-REG compared with those expressing constitutive TDP-43/Raver1, which severely impacted growth in a dose-dependent manner (fig. S10). Thus, *TDP-REGv2:Raver1* vectors can rescue endogenous CEs whilst avoiding toxicity due to constitutive TDP-43/Raver1 protein expression.

## Discussion

We have developed TDP-REG, which enables disease-specific spatial and temporal regulation of gene therapy expression by exploiting the predictable splicing changes induced by TDP-LOF. Given that TDP-LOF is a hallmark of ALS, FTD, and other common neurodegenerative diseases including Alzheimer’s and LATE, TDP-REG could have broad utility in the treatment of these disorders. Disease-induced activation of gene therapies at single-cell resolution could help mitigate the potential risks of permanent transgene expression in patients (14). Furthermore, in at-risk individuals carrying causal genetic variants of ALS, the spatial and temporal specificity of TDP-REG could allow the therapeutics to be delivered at the pre-symptomatic stage, lying dormant until the very first stages of TDP-43 pathology are detected. Additionally, TDP-REG can be used during the preclinical phase of drug development as a real-time readout for TDP-43 pathology in cells or even live animals.

TDP-REGv1 involves the fusion of a transgene to an upstream regulatory module, featuring a modified CE sequence derived from an existing human gene. This approach has several benefits: as a modular design, different transgenes can be controlled by the same upstream regulatory module. Furthermore, TDP-REGv1 is based on a well-validated endogenous CE which is detected in patients in a disease-specific manner.

In contrast, TDP-REGv2 uses synthetic splicing sensors that are embedded within the transgene sequence itself. This addresses several limitations with TDP-REGv1 and previous approaches (26): it removes the need for a lengthy upstream regulatory region, thus aiding packaging of large transgenes into AAV vectors; it prevents an upstream, unwanted peptide being expressed and released into the cell; and it is highly tunable, meaning that splicing characteristics can be optimized for the specific transgene being delivered.

Splicing regulation is highly complex and difficult to predict with conventional algorithms. During the development of TDP-REGv2, we created an algorithm, *SpliceNouveau*, which leverages the power of deep-learning splicing prediction and rational design principles to optimize vectors, helping ensure they are spliced in the desired manner. Although SpliceAI was not directly trained to predict cryptic splicing, we found that its prediction scores correlate with cryptic splice site usage. Compared to using high-throughput screens to find sequences with desired splicing characteristics (27), use of *SpliceNouveau* means only a handful need to be experimentally validated. Using *SpliceNouveau*, we successfully generated cryptic cassette exons, and also cryptic alternative splice sites, by computationally designing competitor alternative splice sites that are used preferentially when TDP-43 is present. The flexibility and high success rate of *SpliceNouveau*-designed vectors therefore represents a major step forward in splicing-regulated vector technology (26, 27).

Overall, this study demonstrates how cryptic splicing, ostensibly a driver of ALS and FTD progression, can be reverse-engineered and exploited to enable exceptionally targeted therapeutic protein expression. This approach, which is compatible with conventional AAVs that have previously been approved for gene therapy, can readily be adapted for different transgenes, including tuning of their maximal expression and sensitivities to TDP-43 depletion. This will minimize the risk of toxic side effects and thus improve the chance of obtaining measurable improvements during clinical trials, reducing danger for patients, and increasing incentives for drug developers.

## Materials and methods

### Data visualization

All data visualization was performed using R. A markdown script containing code for generating all figures is available at 10.5281/zenodo.11576269.

### Cell culture

SK-N-BE(2) and SH-SY5Y cells were grown in DMEM/F12 (Thermo Fisher Scientific) with 10% FBS (Gibco; Thermo Fisher Scientific). HEK293T cells were grown in DMEM Glutamax (Thermo Fisher Scientific) with 10% FBS (Gibco; Thermo Fisher Scientific).

Transfections were performed with Lipofectamine 3000 (Thermo Fisher Scientific), using 20 μl of Lipofectamine and 20 μl of P3000 reagent per microgram of DNA diluted in Opti-Mem (Thermo Fisher Scientific), following the manufacturer protocol.

A clonal SK-N-BE(2) line expressing a doxycycline-inducible shRNA against TDP-43 was generated by transducing cells with SmartVector lentivirus (V3IHSHEG_6494503), followed by selection with puromycin (1 μg ml−1) for one week.

Polyclonal piggyBac lines were generated by co-transfecting the relevant piggyBac vector (backbone from Addgene plasmid #175271) with a vector expressing hyperactive piggyBac transposase (28). A 3:1 ratio of transposase vector to expression vector was used. Selection was performed for at least two weeks in 10 μg/ml blasticidin; a control transfection without the transposase expression vector was performed in parallel, to ensure total cell death of transiently transfected cells after selection.

### Western blotting

Adherent cells were washed with PBS, then lysed in RIPA buffer (25 mM Tris-HCI Buffer pH 7.5, 150 mM NaCl, 1% NP-40, 1% sodium deoxycholate, 0.1% sodium dodecyl sulphate). DNA was sheared via sonication using a Bioruptor Pico device. Samples were loaded onto NuPAGE 4-12% Bis-tris gels (Thermo Fisher Scientific) and transferred to a methanol-activated PVDF membrane using a Mini Trans-Blot Cell (BioRad). Membranes were blocked in 5% fat-free powdered milk in PBS-T buffer (0.2% Tween-20). Primary and secondary incubations were 90 min at room temperature, or overnight at 4°C. Chemiluminescence signal was detected by adding HRP (horseradish peroxidase) substrate (Cytiva, RPN2109). All antibodies used are listed in Supplementary Table S1.

For His-tag pulldown, the Dynabeads His-Tag Isolation and Pulldown kit (Thermo Fisher Scientific, 10103D) was used as per the manufacturer’s instructions. Cells were lysed using lysis buffer (50 mM sodium phosphate pH 8.0, 1% Triton X-100, 50 mM NaCl in distilled water with cOmplete EDTA-free protease inhibitor (Roche)), then combined with an equal volume 2X Binding/Wash Buffer (50 mM sodium phosphate pH 8.0, 600 mM NaCl and 0.02% Tween-20 in distilled water). Following this, the solution was mixed with Dynabeads magnetic beads and incubated at room temperature for 5 min. After placing the sample on a magnet for 2 min, the supernatant was aspirated and discarded. Subsequently, four washes were performed using 1X Binding/Wash Buffer (diluted to 1x with distilled water), ensuring each wash included thorough resuspension of the beads. Finally, the protein was eluted with His-Elution Buffer (300 mM imidazole, 50 mM sodium phosphate pH 8.0, 300 mM NaCl and 0.01% Tween-20 in distilled water). The eluted sample was mixed with NuPage LDS Sample buffer (4x) (Thermo Fisher Scientific) and the western blot was performed as described above.

Quantifications of STMN2 were performed using the ImageJ/Fiji gel analysis tool. Quantifications from three polyclonal lines for each construct were used. The ratio of STMN2 to tubulin was first calculated for each lane (‘normalized STMN2’). The ratio of normalized STMN2 (the ratio of ratios) between the untreated sample and the TDP-43 knockdown sample for each line was then calculated.

### Cloning

dsDNA fragments were ordered from IDT as GBlocks or EBlocks. PCRs were performed using high-fidelity DNA polymerases (Phusion HF 2x Master Mix or Q5 2x Master Mix; NEB). Plasmid backbones were linearised either via inverse PCR or restriction enzyme digestion. Gibson assembly was performed with 2x HiFi Assembly Master Mix (NEB). Transformation of DNA was performed with Stbl3 bacteria (ThermoFisher Scientific); for the transformation of Gibson assembly products, the reaction mixtures were first purified using SPRI beads to avoid toxicity (Mag-bind TotalPure NGS; Omega-Bio-tek). Ligations were performed using T4 DNA ligase (NEB), following phosphorylation with T4 PNK kinase (NEB). All PCR products that used a plasmid as a template were treated with DpnI (NEB) before downstream steps. All relevant sequences were confirmed either by Sanger sequencing (Source Bioscience or Genewiz) or Nanopore sequencing (Plasmidsaurus or Full Circle). The full plasmid sequences of all plasmids generated in this study are available in Supplementary Table S4.

pPB-EF1a-MegaGate-DD-Blast, which was used as the backbone for piggyBac vectors, was a gift from George Church (Addgene plasmid # 175271 ; http://n2t.net/addgene:175271 ; RRID:Addgene_175271) (29). pCMV-PEmax, which was used as the basis for CE-containing Prime Editing vectors, was a gift from David Liu (Addgene plasmid # 174820 ; http://n2t.net/addgene:174820 ; RRID:Addgene_174820) (24); a Tri-Flag-Tagged version was generated via Gibson assembly. pU6-tevopreq1-GG-acceptor, which was used for cloning pegRNAs, was a gift from David Liu (Addgene plasmid # 174038 ; http://n2t.net/addgene:174038 ; RRID:Addgene_174038) (30). A modified version of the 12QN plasmid was generated via Gibson assembly with the same amino acid sequence as published (20).

For the *AARS1*-based plasmids (TDP-REGv1), the cryptic exon sequence, plus short flanking sequences near each of the four relevant splice sites, including the TG-repeat region expected to confer TDP-43-mediated regulation, were fused to create a shortened minigene (hg38 chromosome 16 coordinates: 70276506-70276425, 70272982-70272716 and 70272121-70271940, all on the reverse genomic strand). An ‘AA’ dinucleotide was added within the UG-repeat to enable gene synthesis. An extra adenosine was added to the cryptic exon to enable frame-shifting and several point mutations were added to reduce the creation of unintended splice sites, as predicted by SpliceAI.

### Cell imaging and quantification

Cells were prepared as described above. 96 well plates were seeded with 10,000 cells per well, then transfected as described above using 100 ng of DNA per well. After 52 hours, cells were imaged using an Incucyte microscope (Sartorius). Images were then analyzed using CellProfiler (31): briefly, red objects were identified using an adaptive threshold (“Robust Background” method), then the total intensity of red signal within these objects was calculated. The mean and standard deviation of the four images for each well, followed by the means of each condition (each construct +/- doxycycline) were calculated, and the ratio +/- doxycycline was calculated.

### Quantification of cryptic *AARS1* expression in published RNA-sequencing

Publicly available cell line data were aligned using the pipeline described in (7) - briefly, samples were aligned to the GRCh38 genome build using STAR (v2.7.0f) (32) with gene models from GENCODE v31 (33). NYGC ALS consortium RNA-seq data were processed and categorized according to TDP-43 proteinopathy as previously described (7, 34, 35). Counts for specific junctions were tallied by parsing the STAR splice junction output tables using bedtools. Splice junction parsing pipeline is implemented in Snakemake version 5.5.4 and available at: https://github.com/frattalab/bedops_parse_star_junctions. For quantifying the PSI of the *AARS1* cryptic exon, we extracted the counts from the STAR splice junction output tables using bedtools (36) for spliced reads mapping to the following coordinates:

chr16 70272882 70276486 AARS1_novel_acceptor -

chr16 70271972 70272796 AARS1_novel_donor -

chr16 70271972 70276486 AARS1_annotated -

We calculated the percent spliced in (PSI) as:

$$\text{PSI}=\frac{\;inclusionreads\;}{\;inclusion\;reads\;+\;exclusionreads}\times 100$$

### SpliceNouveau algorithm

Briefly, the algorithm takes a number of parameters as input; as a minimum, the amino acid sequence to be encoded, and the type and position of the cryptic exon/cryptic splice site, are required. The algorithm then initializes a coding sequence for the given amino acid sequence (or uses an initial sequence provided by the user), and uses SpliceAI (17) to assess its predicted splicing behavior, which is compared to the desired, “ideal” set of splicing predictions. The ideal splicing predictions will vary depending on the type of vector being designed (depending on the user command supplied), but in all cases the constitutive splice sites would ideally have very high scores (close to 100%) whereas the cryptic splice sites could vary from low scores (1-10%) to very high scores (close to 100%) depending on the desired level of cryptic splicing; for a single intron vector with alternative splicing, it may be beneficial to ‘balance’ the relative strengths of the cryptic splice site and the competing splice site; to reduce the risk of off-target splicing, the ideal scores of all other positions in the sequence is 0%. Mutated versions (mutations within the coding sequence are always synonymous) of the sequence are then created and their splicing predictions are calculated. The mutant sequences with the highest “fitness” (the sequence with a splicing prediction most closely matching the ideal sequence, calculated by finding the negative sum of absolute differences between the desired and predicted splicing scores at each position) are retained and used as the basis for subsequent rounds of *in silico* mutagenesis. As such, the process resembles a “directed evolution” approach but is performed *in silico*. For single intron designs (excluding intron retention), a competitor splice site is also generated, at a suitable position determined by the algorithm. Additionally, for single introns with alternative 3’ splice sites, the coding sequence upstream of the competitor can be automatically modified with synonymous codons to create a high density of pyrimidines, forming an alternative polypyrimidine tract within the coding sequence, to help generate the competitor splice site. To enable TDP-43-mediated repression of the presumed cryptic splice sites, high densities of UG dinucleotides were specified near the relevant splice sites in all cases. The full sequences of all resulting vectors are available in Supplementary Table S4.

### Screening of synonymous Cas9-encoding variants

A long ssDNA oligo containing degenerate bases at the relevant codon wobble positions was ordered from IDT as an Ultramer (“cas9_ultramer”). This oligo was then converted to dsDNA via PCR. The *AARS1*-based reporter was linearized via inverse PCR, deleting the region corresponding to the cas9_ultramer sequence. The two PCR products were combined with Gibson assembly. The Gibson assembly product was purified using SPRI beads and the whole region relevant to splicing (the candidate CE, its flanking introns, and their flanking exonic sequences) was then amplified via PCR. In parallel, pTwist-CMV was linearised using a primer containing a random barcode. The barcoded linearized vector and PCR-amplified library of candidate CE sequences were then combined via Gibson assembly (HiFi Assembly Master Mix; NEB). Following purification with SPRI beads, the mixture was transformed into Stbl3 bacteria (Thermo-Fisher Scientific), which, following recovery, was transferred directly to ampicillin-LB (without a plating step).

Cas9_ultramer: 5’-

GTGTGTGTGTCACCCAGRCTNTCNCGNAARCTNATHAAYGGNATHCGNGAYAARCARTCNGGNAARACNA THCTNGAYTTYCTNAARTCNGAYGGNTTYGCNAAYCGNAAYTTYATGCARCTNATHCAYGAYGAYTCNCT NACNTTYAARGARGAYATHCARAARGCNCARGTATGCATCACCCCC-3’

The library of plasmids was purified, then transfected into SK-N-BE(2) cells with or without doxycycline-inducible TDP-43 knockdown. RNA was purified then reverse transcribed using a reverse transcription primer featuring a UMI (unique molecular identifier); this was then amplified via PCR using primers with Illumina-compatible overhangs. The resulting PCR products were sequenced via Illumina sequencing. The reads were analyzed using custom Python and R scripts (code available at 10.5281/zenodo.11576269).

### Nanopore analysis

Targeted RT-PCR was performed using vector-specific primers. Barcoding was performed either using custom barcoded primers, or via barcode ligation with kit SQK-NBD114.24. Basecalling was performed using Guppy v6.0.1, with the relevant “SUP” (super-accuracy) model. Demultiplexing was performed using the demultiplex_nanopore_barcodes.py function from nano_tools v0.0.1. Alignment was performed using Minimap2 (v2.1) (37). Pileups were generated using the perform_enhanced_pileup.py function from nano_tools v0.0.1. Splicing analysis was performed by extracting splice junctions from reads using the extract_splice_junctions_from_bam.py function from nano_tools v0.0.1, followed by analysis with custom R scripts (available in 10.5281/zenodo.11576269).

### Generation of AAVs

Vectors were generated using Gibson assembly and full nanopore sequencing (Plasmidsaurus) was used to validate the sequences; all vectors featured the human Synapsin promoter. rAAVs were produced by triple transduction of HEK-293T cells essentially as described in Challis et al. (38), with the following exceptions: 2-4 15 cm dishes were used and after the PEG precipitation samples were resuspended in 4 ml, 3 ml chloroform was added, mixed for 2 min by vortexing, and centrifuged at 3000 g for 20 min and the aqueous layer was loaded on iodixanol gradients in a Type 70.1 rotor and centrifuged for 2 h at 52000 RPM. The rAAV sample was collected and buffer exchanged with 1x PBS 5% Sorbitol 0.1 M NaCl (0.25 M NaCl final). Addgene plasmid #103005 was used for AAV production (39). Titers were between 1.0E14 and 3.1E14 genome copies per ml.

### Mice

All animal care and experimental procedures were performed in accordance with animal study proposal ASP23-003 approved by the National Institute of Child Health and Human Development Animal Care and Use Committee. TDP-43^Fl/wt^ (*Tardbp^tm1.1Pcw^*/J) mice obtained from Dr. Philip Wong at Johns Hopkins University (Jax stock No. 017591) were crossed to homozygosity then crossed to the Chat-IRES-Cre::deltaNeo line (***Chat^tm1(cre)Lowl^***/J);Jax Stock No. 031661), in which the neomycin resistance cassette was removed to avoid ectopic expression sometimes observed in the ChAT-IRES-Cre line. This produced male TDP-43^Fl/wt^;Chat-Cre^+/+^ breeders which were crossed to female TDP-43^Fl/Fl^ mice to generate both TDP-43^Fl/Fl^;Chat-Cre^+/wt^ and TDP-43^Fl/wt^;Chat-Cre^+/wt^ that were used in these experiments. The positive control mScarlet AAV was injected into mice with a Sun1-tag (TDP43 fl/fl;Sun1-GFP +/+), which were generated by crossing the previously described TDP43 fl/fl mice to CAG-Sun1/sfGFP mice (B6;129-*Gt(ROSA)26Sor^tm5(CAG-Sun1/sfGFP)Nat^*/J; Stock No: 021039). The genetic background was C57Bl/6J for all animals. All animal ages are listed in Supplementary Table S2.

### Intracerebroventricular Injections

A 10 μl Hamilton syringe (65460-06) with a 33G needle (65461-02) was loaded with up to 10 μl of undiluted virus and placed on a syringe pump (KD Scientific 78-0220). Postnatal day 0-2 pups were anesthetized on ice for approximately 1 minute. After anesthesia, pups were placed on a sterilized mobile surface and advanced such that the Hamilton syringe penetrated the left ventricle. 1 μl of virus was delivered at 1 μl/min into the left ventricle, approximately 1 mm lateral from the sagittal suture. The syringe was kept in place for approximately 30 seconds after the injection removal to minimize backflow. Pups were placed on a heating pad to recover before being returned to their dam in the home cage. Injection details are in Supplementary Table S2.

### Tissue Preparation and Sectioning

Mice (age 3-7 weeks) were anesthetized with I.P. injections of 2.5% avertin and transcardially perfused with 10mL of 1X PBS followed by 10mL of 4% paraformaldehyde. Spinal cords were dissected and placed in 4% paraformaldehyde overnight before being placed in a 30% sucrose in PBS cryopreservation solution. After 24 hrs in cryopreservation solution, lumbar spinal cords were embedded in O.C.T (Tissue-Tek) and frozen. Frozen blocks were sectioned into 16 μm-thick coronal slices onto positively charged slides using a Leica CM3050 S Research S Cryostat. Slides were stored at -80°C for up to 2 weeks.

### Immunostaining of Tissue

Slides were removed from -80°C and thawed to room temperature, then washed in 1X PBS before being placed in citrate buffer (pH 6.0) for antigen retrieval. The solution and slides were microwaved for 45 seconds (until light boil) and allowed to cool back to room temperature. Tissue was then permeabilized in 0.1% Triton-X100 in 1X PBS (PBSTx), then blocked in 5% normal donkey serum in 0.1% PBSTx. Primary antibodies were diluted in 0.5% normal donkey serum in 0.1% PBSTx and incubated overnight at 4°C. Slides were then washed in 0.1% PBSTx and incubated for 1 hr in secondary antibody (ThermoFisher) diluted in 0.1% PBSTx. After a final 1X PBS wash, slides were coverslipped with Prolong Diamond (ThermoFisher P36961) and dried overnight at room temperature in the dark before being stored at 4°C. Primary antibodies: Rat anti-TDP-43 (Biolegend #808301, 1:3000), Rabbit anti-RFP (Rockland #600-401-379, 1:100), Guinea Pig anti-VAChT (Synaptic Systems #139105, 1:500).

### Imaging and Analysis

Up to 6 slides were loaded simultaneously onto the Olympus VS200 slide scanner for imaging. Slides were imaged at 20X with 5 z planes, 2 μm apart with the following filer cubes: DAPI, FITC, TRITC, and Cy5. For motor neuron counts, maximum intensity projections were used. Briefly, ROIs were drawn around motor neurons based on VAChT and DAPI by an investigator blind to genotype. To determine RFP positive vs negative motor neurons, the brightness was adjusted so that no RFP was detectable in background spinal cord regions or in between motor neurons. Any motor neurons that still had detectable RFP were considered positive while those with background levels were considered negative.

### Cytoplasmic aggregation

A plasmid encoding SNAP-TDP-43-12QN with an L207P mutation in the human TDP-43 sequence was generated; the sequence of the TDP-43 and 12xQN repeat was identical to a published study (18). For confocal imaging, TDP-REGv2 mScarlet reporter construct #7 was co-transfected with SNAP-TDP-43-12QN, at a 1:3 mass ratio of mScarlet reporter:SNAP/TDP plasmid. Cells were plated on Matrigel-coated Ibidi 8-well microscopy dishes, then transfected after 24 hours. Three hours prior to fixation, SNAP-Cell Oregon Green (NEB) prepared in DMSO (in accordance with the manufacturer recommendations) was diluted into growth media (1:2,000 v/v) and the original media was replaced with this media. 60 min prior to fixation, the SNAP-Cell Oregon Green media was replaced with normal growth media. HEK293T cells were fixed 28 hours after transfection, whereas SK-N-BE(2) cells were fixed 50 hours after transfection, to compensate for their lower protein expression levels; fixation was performed in 2% PFA (methanol-free) diluted in PBS for 20 min at room temperature. Cells were permeabilized with 0.1% Triton X100 diluted in PBS for 10 min at room temperature. Blocking was performed for one hour with 0.1% Tween 20 and 2% W/V bovine serum albumin (BSA) diluted in PBS. Primary antibody (TDP-43 antibody; Proteintech) was diluted 1:500 into PBS with 0.1% Tween 20 and cells were incubated overnight at 4°C. After three washes with PBS-Tween 20, secondary antibody (anti-Rabbit IgG conjugated to Alexa-fluor 647; Abcam) was diluted into PBS with 5% w/v BSA and 0.05% Tween 20, and incubation was performed for 60 min at room temperature. Finally, samples were washed and incubated with DAPI for nuclear staining. Imaging was performed on a Zeiss 880 Inverted Confocal using a 63x oil objective lens. To reduce bleed-through from the far-red channel into the red channel, a narrow wavelength cut-off was used for the native mScarlet signal of approximately 575-595 nm.

For imaging with the Incucyte, HEK293T cells were plated into a 96 well plate. The following day, they were transfected with 70 ng of SNAP-12QN-TDP-43, SNAP-TDP-43(Wild-type) or SNP-only, 20 ng of TDP-REGv2:mScarlet reporter plasmid #7 and 10 ng of mGreenLantern plasmid (as a transfection control) (40, 41). Media was changed 24 hours after transfection. Imaging on an Incucyte S5 was performed 48 hours after transfection.

### Cre recombinase with multiple cryptic exons

Vectors encoding Cre recombinase containing 1, 2 or 3 cryptic exons were created via Gibson assembly. Vectors were transfected into SK-N-BE(2) cells with/without doxycycline-inducible TDP-43 knockdown. 48 hours after transfection, RNA was extracted and targeted Nanopore sequencing was performed. Reads were analyzed with nano_tools (v0.0.1) and analysis plots were generated in R (code available at 10.5281/zenodo.11576269).

### Testing TDP-REG specificity using shRNAs

shRNA sequences were designed by finding the top consensus sequences as predicted by two algorithms (42, 43). The selected shRNAs were cloned into a mir30E locus in a vector via Gibson assembly; each plasmid also encoded blasticidin resistance. SK-N-BE(2) cells were co-transfected with TDP-REGv2 mScarlet construct #10 (30 ng) and one shRNA plasmid (70 ng) using Lipofectamine 3000, following the manufacturer’s recommendations. 24h after transfection, media was replaced with fresh media containing 5 μg/ul blasticidin. Five days after transfection, cells were imaged using an Incucyte S3, using 4x magnification. Fluorescence levels were analyzed using R (code available at 10.5281/zenodo.11576269).

### Prime editing

*SpliceNouveau* was used to design a cryptic exon within the pCMV-PEMax vector. The vector was transfected in SK-N-BE(2) cells with or without TDP-43 knockdown and the splicing of the construct was analyzed via RT-PCR. Primers are listed in Table S3.

The PrimeDesign web tool was used to design the pegRNA and nicking sgRNA (44).

The pegRNA used had sequence 5’-GTAAAAGCATGGATGGAGAGAGTTTTAGAGCTAGAAATAGCAAGTTAAAATAAGGCTAGTCCGTTATCAA CTTGAAAAAGTGGCACCGAGTCGGTGCATGGACTCACGCATCTCTCCATCCATGCCGCGGTTCTATCTAG TTACGCGTTAAACCAACTAGAATTTTTTT-3’

The sgRNA used had sequence 5’-GAAACACCGTGGGGATAAGAGTTCTTTCCGTTTTAGAGCTAGAAATAGCAAGTTAAAATAAGGCTAGTCC GTTATCAACTTGAAAAAGTGGCACCGAGTCGGTGCTTTTTT-3’

pCMV-PEMax or a version containing a cryptic exon were transfected into SK-N-BE(2) cells with or without doxycycline-inducible TDP-43 knockdown. 600 ng of prime editing vector was used, in addition to 200 ng of pegRNA, 100 ng of nicking sgRNA and 100 ng of a plasmid expressing mScarlet and the blasticidin resistance gene. 24 hours after transfection, the media was changed and supplemented with 10 μg/ml blasticidin to select for transfected cells. After an additional 48 hours, the cells were subcultured, and after six days total the samples were harvested and gDNA was purified. gDNA was amplified via PCR and amplicons were analyzed by Nanopore sequencing. Pileups were generated using the perform_enhanced_pileup.py function from nano_tools v0.0.1. The fraction of reads containing the expected edit was calculated.

### Luciferase analysis

A modified Gaussia luciferase amino acid sequence, in which the methionines are replaced to improve resistance to oxidation, was reverse-translated and optimized by SpliceNouveau, including internal cryptic exons. Vectors were transfected into SK-N-BE(2) cells with or without doxycycline-inducible TDP-43 knockdown. Luciferase activity was measured by extracting an aliquot of cell media and mixing with Pierce™ Gaussia Luciferase Glow Assay Kit (Thermo Fisher Scientific) following the manufacturer protocol. Splicing was analyzed via targeted Nanopore sequencing, using the approach described above.

### Design of TDP-43/Raver1 expression vectors

The TDP-43/Raver1 protein sequence used for all experiments was:

MGPKKKRKVEDPGG*PAAKRVKLD*GG**YPYDVPDYA**GGM**SEYIRVTEDENDEPIEIPSEDDGTVLLSTVTAQ FPGACGLRYRNPVSQCMRGVRLVEGILHAPDAGWGNLVYVVNYPKDNKRKMDETDASSAVKVKRAVQKTS DLIVLGLPWKTTEQDLKEYFSTFGEVLMVQVKKDLKTGHSKGFGFVRFTEYETQVKVMSQRHMIDGRWCD CKLPNSKQSQDEPLRSRKVFVGRCTEDMTEDELREFFSQYGDVMDVFIPKPFRAFAFVTFADDQIAQSLC GEDLIIKGISVHISNAEPKHNSN**LPPLLGPSGGDREPMGLGPPATQLTPPPAPVGLRGSNHRGLPKDSGP LPTPPGVSLLGEPPKDYRIPLNPYLNLHSLLPSSNLAGKETRGWGGSGRGRRPAEPPLPSPAVPGGGSGS NNGNKAFQMKSRLLSPIASNRLPPEPGLPDSYGFDYPTDVGPRRLFSHPREPTLGAHGPSRHKMSPPPSS FNEPRSGGGSGGPLSHF*

In the above sequence, the SV40 NLS is underlined, the c-Myc NLS is underlined and in italics, the HA-tag sequence is in bold, the TDP-43 sequence is in bold and underlined, and the C-terminal Raver1 sequence is in italics. The 2FL mutation changed the sequence HSKGFGF within RRM1 to HSKGLGL.

*SpliceNouveau* was used to design constructs with internal cryptic exons within the region encoding TDP-43 within the TDP-43/Raver1 sequence above. For initial screening of their cryptic exon properties, the designed sequences were cloned into a mammalian expression vector (pTwist-CMV) containing the 2FL mutation. These were then transiently transfected into SK-N-BE(2) cells with or without doxycycline-induced TDP-43 knockdown. RNA was extracted, reverse transcribed and RT-PCRs were performed to analyze inclusion of the synthetic TDP-43-encoding cryptic exons (primers listed in Table S3), which was aided by the use of a QIAxcel Advanced machine (QIAGEN).

### Rescue of endogenous cryptic splicing with TDP-43/Raver1

The piggyBac system was used to make SK-N-BE(2) cell lines with constitutive EF1A promoters driving expression of constitutive (without a cryptic exon) or two cryptic exon-containing TDP-43/Raver1 constructs, or mScarlet; a PGK promoter drove expression of the blasticidin resistance gene (29). Note that these lines were made using the clonal line that featured the doxycycline-inducible TDP-43 shRNA, enabling doxycycline-inducible TDP-43 knockdown. Polyclonal lines were produced in triplicate. Each polyclonal line was then plated with or without doxycycline treatment (1000 ng/ml) for six days, then harvested for western blotting or RT-PCRs. Western blotting was performed as described above.

RT-PCRs for *UNC13A, STMN2* and *AARS1* were performed via reverse transcription with Superscript IV (Thermo Fisher Scientific) followed by PCR using either Q5 2x Master mix (NEB) or One-Taq Quickload 2x Master Mix (NEB). *UNC13A* and *AARS1* PCRs used two primers, whereas *STMN2* used two reverse primers because the STMN2 cryptic exon induces premature polyadenylation; primer sequences are listed in Table S3. PCR products were electrophoresed on a QIAxcel advanced system. Raw data was exported and analyzed in R using the QIAxcelR package (v0.1) (https://github.com/Delayed-Gitification/QIAxcelR; 10.5281/zenodo.11576269).

### Growth competition assay

The piggyBac system was used to generate SK-N-BE(2) cells with stably-integrated, doxycycline-inducible expression of TDP-43/Raver1 fusion, either constitutive or with an internal cryptic exon, or mTag-BFP2 blue fluorescent protein (45). Cell lines were generated in triplicate. 100 ng of each vector, plus 400 ng of hyperactive piggyBac transposase, were used per transfection (per well of a 24-well plate). Note that in this case, the SK-N-BE(2) cells did not feature the doxycycline-inducible TDP-43 shRNA cassette.

Following selection of stable cells in 10 μg/ml blasticidin for 20 days, blasticidin was removed and the different cell lines were mixed, again in triplicate. Each mix was placed into three different wells, with 0, 30 or 1000 ng/μl of doxycycline. After three days, the cells were subpassaged. After a further seven days, the cells were lysed in 20 mM Tris-HCl pH 7.5, 0.5 mM EDTA, 1% Trixon X100 and 500 ng/μl proteinase K; samples were incubated at 55°C for 20 min, then 96°C for 5 min.

A multiplex PCR was performed using two pairs of primers: one pair which amplified the three TDP-43/Raver1 vectors, and another pair which amplified the BFP construct. PCR was performed with Q5 2x Master Mix (NEB), using 2.5 μl of lysate into a 25 μl PCR. Primers are listed in Table S3. The samples were then purified and barcoded Nanopore libraries were prepared using the Native Barcoding 24 kit with R10.4.1 chemistry. The library was sequenced with an R10.4.1 Flongle device and High Accuracy basecalling was used in real-time with Guppy. Reads were then aligned to a “reference genome” consisting of the four constructs with Minimap2 (v2.1) (37), and mapping statistics were calculated by analyzing the resulting bam files in R (scripts available at 10.5281/zenodo.11576269).

### iPSC cell culture and differentiation

For iPSC work, the WTC11 iPSC line (GM25256) harboring stable integration of doxycycline-inducible Tet-on neurogenin-2 (NGN2) and dCas9-BFP-KRAB cassettes at the AAVS1 and CLYBL safe-harbor loci, respectively, were used (46). iPSCs were modified to have a HaloTag for *TARDBP*, and to express TDP-43/Raver1 or mScarlet constructs, as described below.

iPSCs were maintained in E8 Flex Medium (Thermo) in Geltrex (Thermo)-coated plates and passaged with Versene (Thermo) or Accutase (Thermo) when 80% confluent. For induction of iPSCs to i3Neurons, iPSCs were passaged with Accutase (Thermo) and plated onto Geltrex-coated plates with induction media: DMEM/F12 with GlutaMAX (Thermo). 1 x non-essential amino acids (NEAA, Thermo), 2 μg/ml doxycycline hyclate (Sigma), 2 μM XAV939 (Cayman Chemical), 10 μM SB431542 (Cayman Chemical), and 100 nM LDN-193189 (Cayman Chemical). Media was changed daily for three days. For RNA experiments, 12-well plates were coated with poly-D-lysine (10 μg/mL, Gibco) overnight, washed with sterile water, and subsequently coated with laminin overnight (10 μg/mL, Gibco). On the third day, 500k cells were plated per well of the 12-well plate in neuronal media, supplemented with 1x RevitaCell (Thermo): BrainPhys (StemCell Technologies) with 1x N2Max supplement (R&D Systems) 1x N21Max supplement (R&D Systems), 10 ng/mL BDNF (Peprotech), 10 ng/ml GDNF (Peprotech), and 1 μg/mL Laminin (Thermo). 24 hours later, a full media change was performed to remove RevitaCell and to add 300 nM HaloPROTAC-E (University of Dundee) treatment to conditions where TDP-43 knockdown was intended. Differentiated neurons were maintained in neuronal media, and twice-weekly half-media changes were performed. After 14 days, RNA was harvested.

### HaloTag editing of TARDBP

iPSCs were electroporated with 10 μg HaloTag-*TARDBP* homology-directed repair template, 500 pmol Cas12 crRNA (GGAAAAGTAAAAGATGTCTGAAT, IDT) and 20 μg recombinant Cas12 (IDT) using the P3 Primary Cell 4-D-Nucleofector kit (Lonza) and 4D-Nucleofector X unit (Lonza) using the CA-137 program. After electroporation iPSCs were plated in E8 Flex Medium (Thermo) supplemented with 1x RevitaCell (Thermo) and 1x Alt-R HDR Enchancer V2 (IDT) for 24 hours and then expanded in E8 Flex Medium. Cells were labeled with HaloTag-TMR ligand (Promega) and positive clones were selected for genotyping by PCR.

### Generation of RAVER iPSC lines

1 million cells were plated per well of a Geltrex-coated six-well plate in E8 Flex media containing 1x RevitaCell. Two hours later, media was changed to E8 Flex without RevitaCell, and iPSCs were transfected with 2.25 mg piggyBac plasmid for TDP-REGv2:TDP-43/Raver1 #6 or #9, or mScarlet control, using Lipofectamine Stem reagent (Thermo Fisher Scientific) along with 0.75 mg hyperactive piggyBac transposase (28). 48 hours post transfection blasticidin selection was started (Sigma) using 6 mg/ml for 24 hours, then 8 mg/ml for 24 hours, and finally 10 mg/ml for two weeks.

### UNC13A synapse quantifications

I3 neuron (500K) and rat astrocytes (50k) were co-cultured on PDL/laminin-coated 18mm coverslips and fixed at D35 for 10min in 4% PFA. Cells were permeabilized in 0.1% triton for 10 mins before incubation with primary antibodies in PBS at RT for 1 hr: 1:500 Munc13-1 (Synaptic Systems - 126-104), 1:1000 synapsin (Synaptic Systems - 106-011). Cells were washed in PBS 3x before incubation with secondary antibodies in PBS at RT for 1 hr: mouse-polyclonal 488 (AlexaFluor) and guinea-pig 647 (Alexafluor). Cells were washed in PBS 3x before mounting to glass slides using Mowiol (PolySciences) containing 1:1000 DAPI (ThermoFisher). Imaging was carried out on a Zeiss 980 airyscan confocal microscope at 63x with a 3x crop. Images comprised 7 Z-stacks of 0.5μm intervals. Images were analyzed using FIJI. ROIs were defined based on synapsin-positive puncta and used to measure the mean UNC13A intensity.

### Testing correlation of SpliceNouveau optimization with vector performance

56 vectors were designed with four different levels of optimization (14 per optimization level), each using the same *SpliceNouveau* command, encoding mScarlet with a single intron and an alternative 3’ splice site design. We specified a strong initial donor splice site motif of ‘CAAGTAAG’, which is among the most common motifs at annotated human donor splice sites. The full command was: python3 SpliceNouveau.py --initial_cds ATGGCGAGAACAATGGTTGCTATGGTGTCCAAGGGTGAAGCAGTCATAAAGGAGTTTATGAGGTTCAAGGTGCACATGGAAGGGTC AATGAACGGACATGAGTTCGAAATTGAAGGGGAGGGCGAGGGCCGCCCCTATGAAGGGACACAAACTGCCAAGCTCAAGGTAACCA AGGGGGGACCCCTACCATTCTCATGGGACATTCTGTCCCCGCAATTCATGTATGGTTCTCGTGCATTCACAAAGCATCCTGCTGAT ATCCCAGACTACTACAAACAATCCTTTCCGGAGGGCTTTAAGTGGGAACGCGTCATGAATTTCGAGGACGGAGGCGCGGTGACGGT CACTCAAGATACCAGCCTAGAGGACGGCACGCTTATTTACAAAGTCAAGCTACGCGGAACGAACTTCCCTCCCGATGGGCCGGTCA TGCAAAAGAAAACAATGGGGTGGGAGGCGTCGACCGAGCGCTTGTACCCCGAGGACGGAGTACTAAAGGGAGATATAAAGATGGCA TTGCGCCTAAAAGACGGGGGACGATACCTGGCCGACTTCAAGACCACCTACAAGGCCAAGAAGCCCGTGCAGATGCCCGGCGCCTA CAACGTGGACCGAAAGCTGGACATCACCAGCCACAACGAGGACTACACCGTGGTGGAGCAGTACGAGAGGAGCGAGGGCAGGCACA GCACCGGCGGCATGGACGAGCTGTACAAGGACTACAAGGACGATGATGACAAA --initial_intron1 GTaagNNNNNNNNNNNNNNNNNNNNNNNNNNNNNNNNNNNNNNNNNNNNNNNNNNNNNNNNNNNNNNNNNNNNNNNNNNNNNNNNN NNNNNNNNNNNNNNNNNNTGTGTGTGTGTGTGTGTGTGAATGTGTGTGTGTGTGTGTGNcAG --ce_start 216 --ce_end 216 --five_utr CGGCCGCTTCTTGGTGCCAGCTTATCAtagcgctaccggtcgccacc --three_utr TAATAAACAAATGGTaagGAAGGGCACATCAATCTTTGCTTAATTGTCCTTTACTCTAAAGATGTATTTTATCATACTGAATGCTA AACTTGATATCTCCTTTTAGGTCATTGATGTCCTTCACCCCGGGAAGGCGACAGTGCCTAAGACAGAAATTCGGGAAAAACTAGCC AAAATGTACAAGACCACACCGGATGTCATCTTTGTATTTGGATTCAGAACTCAGTAAACTGGATCCGCAGGCCTCTGCTAGCTTGA CTGACTGAGATACAGCGTACCTTCAGCTCACAGACATGATAAGATACATTGATGAGTTTGGACAAACCACAACTAGAATGCAGTGA AAAAAATGCTTTATTTGTGAAATTTGTGATGCTATTGCTTTATTTGTAACCATTATAAGCTGCAATAAACAAGTTAACAACAACAA TTGCATTCATTTTATGTTTCAGGTTCAGGGGGAGGTGTGGGAGGTTTTTTAA --ignore_end 470 --aa generate_it --upstream_mut_chance 0.3 --downstream_mut_chance 1 -a 30 --intron1_mut_chance 0.5 -n 3000 --cds_mut_start_trim 160 --cds_mut_end_trim 396 --overwrite --ce_score_weight 3 - -early_stop 500 --target_const_donor 1 --target_const_acc 0.5 --intron1_mut_chance 0.5 --alt_3p --overwrite --alt_position in_exon --alt_3p_end_trim 471 --downstream_mut_n 1 --target_cryptic_acc 0.75 --min_alt_dist 40 --track_splice_scores

These were cloned into a backbone containing dual barcodes (in the 5’ and 3’ UTRs) featuring A, C or T bases in the forward strand (avoidance of G ensures no cryptic splice sites will be created within the barcode. A pool of ~500 plasmids was created, each with a unique barcode combination and with a single vector design. These were transfected into SK-N-BE(2) cells with or without TDP-43 knockdown.

Following RT-PCR, the RNA products from these cells were sequenced using an R10.4.1 Minion flowcell. Additionally, a sample of the plasmid pool was digested using PacI and sequenced in parallel. Following basecalling (Guppy, super-accuracy) the reads were aligned to a ‘genome’ containing all 56 plasmid designs, and barcode pairs were assigned to plasmids using the reads derived from the plasmid DNA. These barcode pairs were then used to assign the RNA-derived reads to plasmids. The mapped reads were analyzed using nano_tools and R (all code available in the R markdown; 10.5281/zenodo.11576269).

### Nanopore analysis of TDP-REVv2:TDP-Raver1 #9 in mouse spinal cord

Mouse spinal cords were removed and flash-frozen in liquid nitrogen. Each cord was lysed in 1 ml of RLT-plus buffer (Qiagen), then homogenized using a glass tissue grinder, followed by centrifugation through a QIAshredder column. The flow-through was processed using the RNeasy Plus kit (Qiagen), following the manufacturer’s protocol. RNA was reverse transcribed using Superscript IV and random hexamers, following the manufacturer protocol, followed by RT-PCRs against the exonic vector sequences flanking the TDP-43-encoding CE. PCR products were purified and sequenced on a R10.4.1 Flongle using the SQK-NBD114.24 kit, basecalled as described, and analyzed using Minimap2 and nano-tools v0.0.1.

## Supplementary Material

Table S2

Table S4

Supplementary Material

## Data Availability

RNA-Seq Data for i3Neurons, SH-SY5Y and SK-N-BE(2) are available through the European Nucleotide Archive (ENA) under accession PRJEB42763. Public data was obtained from Gene Expression Omnibus (GEO): iPSC MNs (Klim et al., 2019)-GSE121569, Appocher SK-N-BE(2)-GSE97262, and HeLa Ferguson-GSE136366. NYGC ALS Consortium RNA-seq: RNA-Seq data generated through the NYGC ALS Consortium in this study can be accessed via the NCBI’s GEO database (GEO GSE137810, GSE124439, GSE116622, and GSE153960). All RNA-Seq data generated by the NYGC ALS Consortium are made immediately available to all members of the Consortium and with other consortia with whom we have a reciprocal sharing arrangement. To request immediate access to new and ongoing data generated by the NYGC ALS Consortium and for samples provided through the Target ALS Postmortem Core, complete a genetic data request form at ALSData@nygenome.org. Targeted sequencing datasets and accompanying Snakemake scripts for data processing are available at https://doi.org/10.5061/dryad.cjsxksnfr. A full R markdown containing code for all data visualization, including small amounts of downstream processing, is available at 10.5281/zenodo.11576269. The full sequences of all vectors used in this study are available in the supplementary materials. A selection of TDP-REG vectors has been made available on Addgene.
