# Supplementary material for: Creation of de novo cryptic splicing for ALS/FTD precision medicine
Source: Science. Author manuscript; Available in PMC 2024 Oct 21. (PMC7616720; doi:10.1126/science.adk2539)
Supplement: Supplementary Material [file EMS199220-supplement-Supplementary_Material.pdf]

Science
AAAS

Supplementary Materials for

Creation of de novo cryptic splicing for ALS/FTD precision medicine

Authors: Oscar G. Wilkins[1,2]*, Max Z.Y.J. Chien[1,2]†, Josette J. Wlaschin[3]†, Simone Barattucci[1], Peter Harley[1], Francesca Mattedi[1], Puja R. Mehta[1], Maria Pisliakova[1,2], Eugeni Ryadnov[1], Matthew J. Keuss[1], David Thompson[4], Holly Digby[2,5], Lea Knez[1,2], Rebecca L. Simkin[1], Juan Antinao Diaz[6], Matteo Zanovello[1,2], Anna-Leigh Brown[1], Annalucia Darbey[1], Rajvinder Karda[6], Elizabeth M.C. Fisher[1], Thomas J. Cunningham[4, 4a], Claire E. Le Pichon[3], Jernej Ule[2,5], Pietro Fratta[1,2]*

Corresponding authors: p.fratta@ucl.ac.uk, oscar.wilkins.18@ucl.ac.uk

The PDF file includes:

Materials and Methods
figs. S1 to S10
Tables S1 to S4 (S2 and S4 are attached separately due to large size)

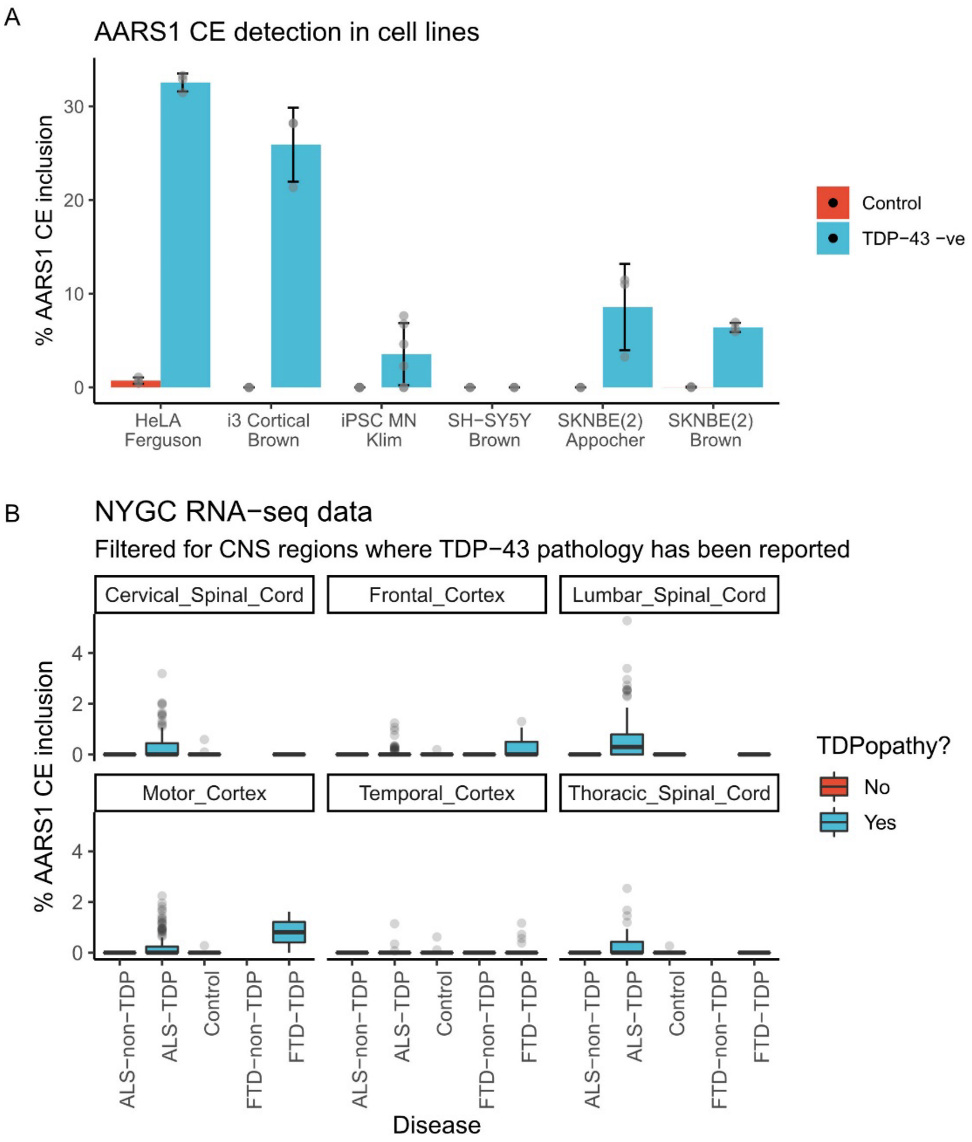

Fig. S1.

A: *AARS1* cryptic exon inclusion percentage for various cell lines in which TDP-43 levels were reduced artificially; error bars show standard deviation. **B:** *AARS1* cryptic exon inclusion for bulk RNA seq from various tissues and patients in the NYGC dataset.

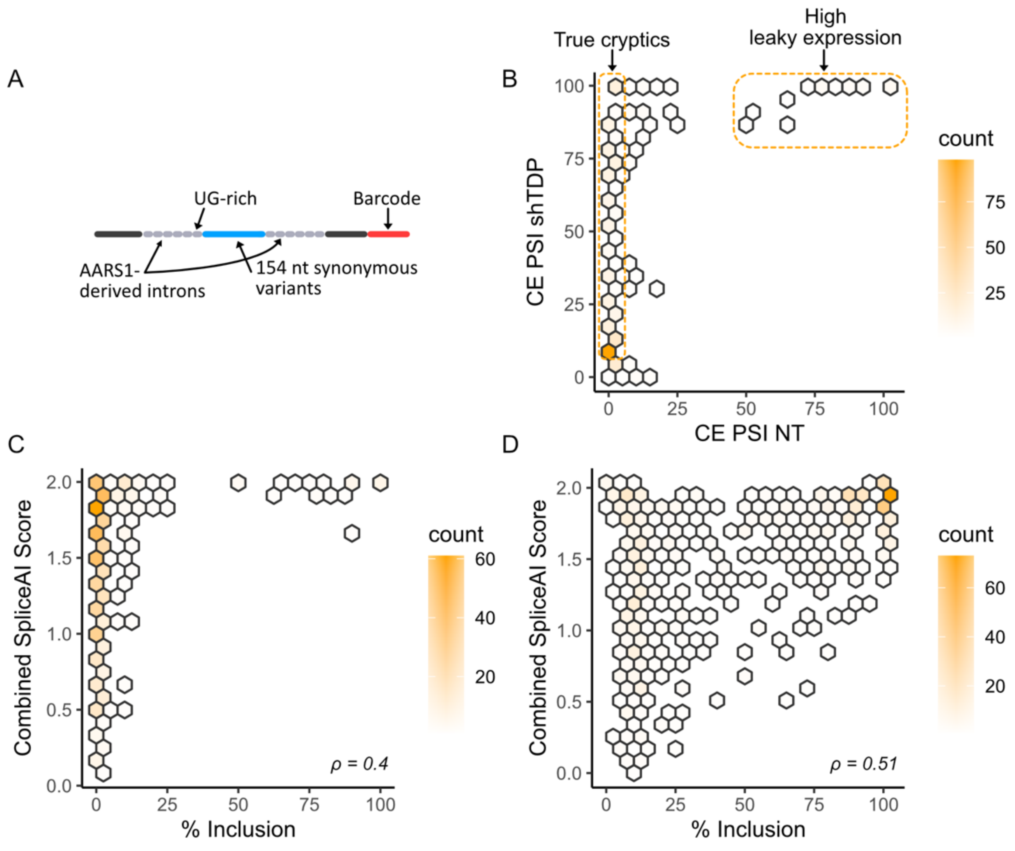

Fig. S2.

A: Schematic of the barcoded library of vectors containing different candidate CE sequences, each encoding the same amino acid sequence (in this case, a fragment of S. pyogenes Cas9) but with different codon optimization. **B:** Heatmap of % CE inclusion for library of Cas9-fragment-encoding cryptic exons in SK-N-BE(2) cells with TDP-43 knockdown versus untreated cells ("NT" = "not treated"). Areas corresponding to "True cryptics" (i.e. those that are only expressed upon TDP-43 knockdown) and "High leaky expression" (i.e. those that are expressed regardless of TDP-43 knockdown) are highlighted. **C:** Heatmap of the SpliceAI combined score of the candidate CE acceptor and donor splice sites against the PSI of each CE in SK-N-BE(2) cells without TDP-43 knockdown (i.e. untreated cells); Spearman correlation shown (bottom)**. D:** Heatmap of the SpliceAI combined score of the candidate CE acceptor and donor splice sites against the PSI of each CE in SK-N-BE(2) cells with TDP-43 knockdown; Spearman correlation shown.

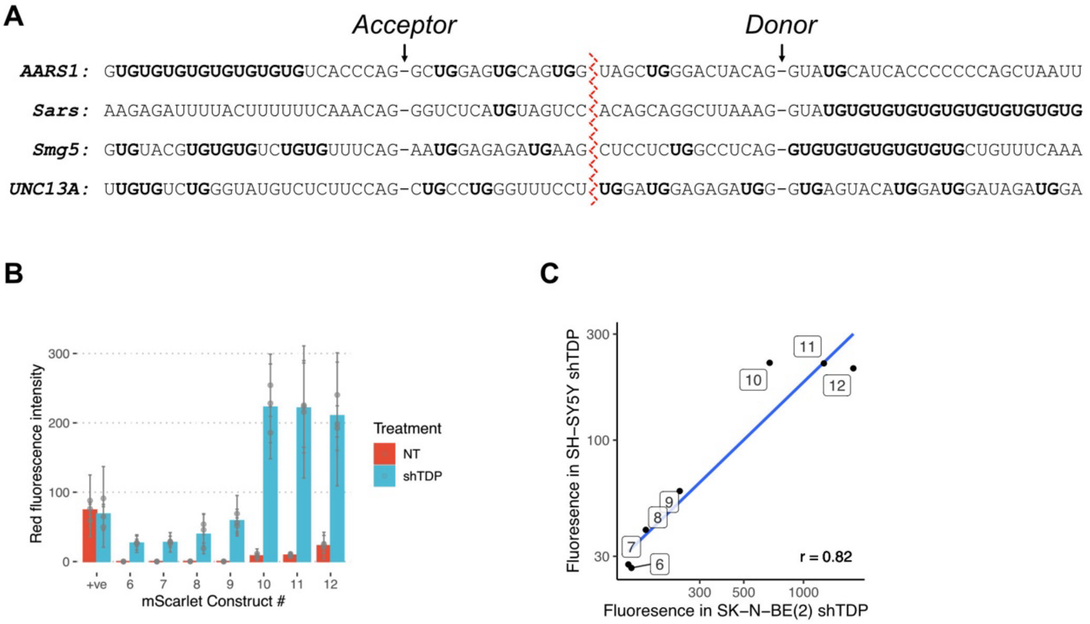

Fig. S3.

A: Diagrams of four endogenous CEs, highlighting the position of UG dinucleotides relative to the splice sites. **B:** Quantification of mScarlet fluorescence from SY-SY5Y cells transfected with mScarlet constructs 6-12; dots show average of each well; error bars show standard deviation within each well. **C:** A comparison of the fluorescence of the seven TDP-REGv2 mScarlet constructs in SY-SY5Y and SK-N-BE(2) cells, with TDP-43 knockdown; Pearson correlation is shown.

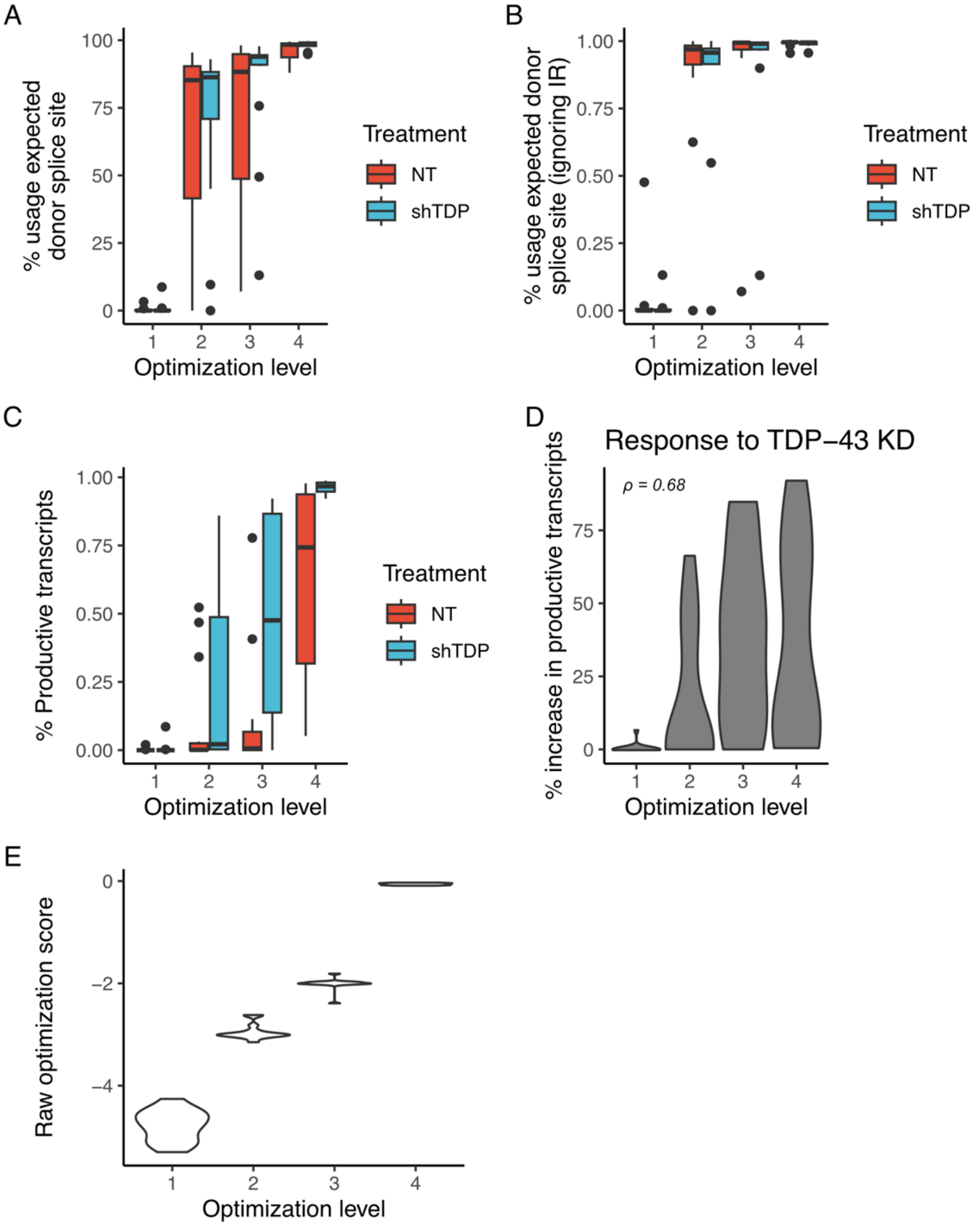

Fig. S4

More optimized vector sequences are more likely to be spliced in the desired manner. **A-B:** % usage of the expected donor splice site for sequences with different optimization levels, as assessed by the *SpliceNouveau* algorithm, including or ignoring intron retention (IR) respectively. **C:** The fraction of productive transcripts (i.e. transcripts that are predicted to express mScarlet) with or without TDP-43 knockdown, separated by optimization level. **D:** % increase in productive transcripts upon TDP-43 knockdown; Spearman's rho value, between optimization level and % increase, is indicated. **E:** Fitness scores for all fourteen vectors of each optimization level.

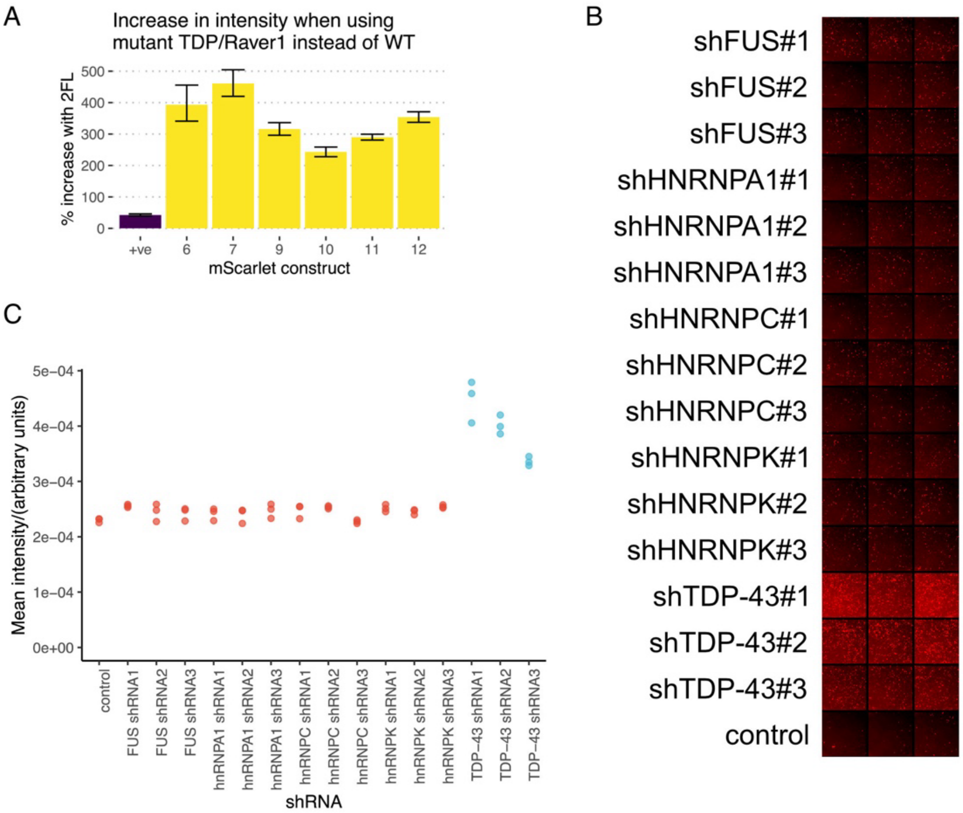

Fig S5: A: Comparison of the fluorescence of six TDP-REGv2 mScarlet constructs and a positive control transfected into SK-N-BE(2) cells with TDP-43 knockdown, when co-transfected with a mutant TDP-43/Raver1 fusion protein (which is unable to rescue splicing) versus when co-transfected with a functional TDP-43/Raver1 fusion protein; error bars show 90% confidence interval as determined by Monte-Carlo methods. **B:** Fluorescence microscopy of SK-N-BE(2) cells co-transfected with a plasmid encoding TDP-REGv2 mScarlet reporter #10 and plasmids encoding the most potent predicted shRNAs against FUS, hnRNPA1, hnRNPC, hnRNPK or TDP-43, or an identical plasmid but without an shRNA sequence. **C:** Quantification of fluorescence from images in part B.

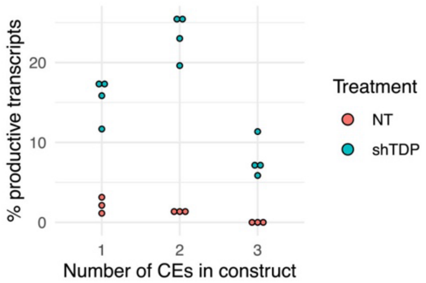

Fig S6: Percentage of productive transcripts, as determined by targeted Nanopore sequencing, from vectors encoding Cre-recombinase with 1, 2 or 3 cryptic exons without (NT) or with (shTDP) TDP-43 knockdown.

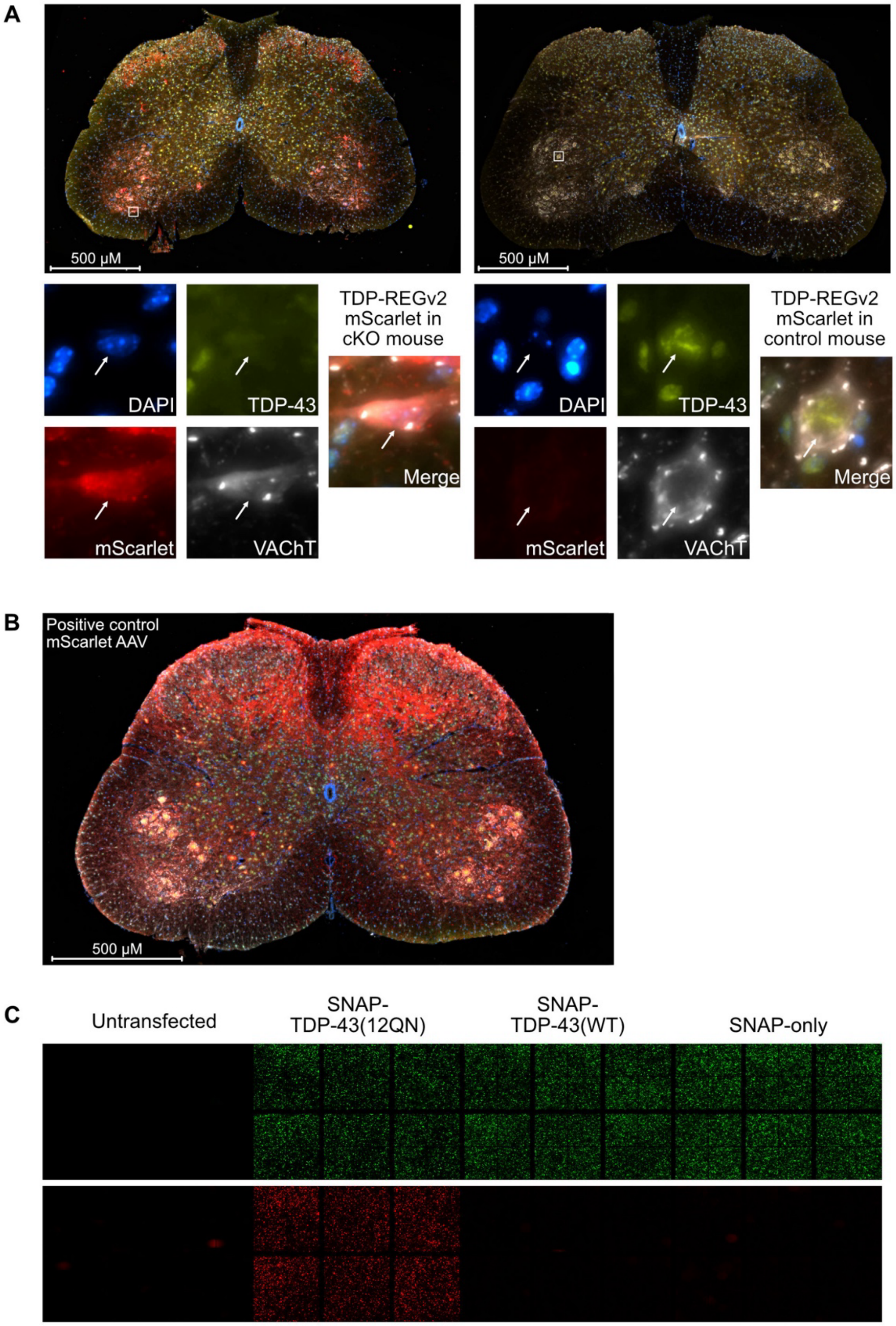

Fig. S7.

A: Fluorescence microscopy of spinal cord sections from TDP-43 cKO or control mice (left and right respectively) injected with a TDP-REGv2 mScarlet AAV (construct #7). Magnifications of two representative motor neurons are shown below; locations are shown in the full images by

white boxes. Blue = DAPI, Yellow = TDP-43, White = VaChT, Red = mScarlet). B: Representative fluorescence microscopy image of a spinal cord section from a control mouse injected with a positive control mScarlet AAV (i.e. without TDP-REG); coloring is the same as Part A. **C:** Fluorescence microscopy images of green (top) and red (bottom) fluorescence in cells co-transfected with the vectors encoding TDP-REGv2:mScarlet #7 the proteins indicated above plus an mGreenLantern-encoding transfection control; 6 replicates per condition were used.

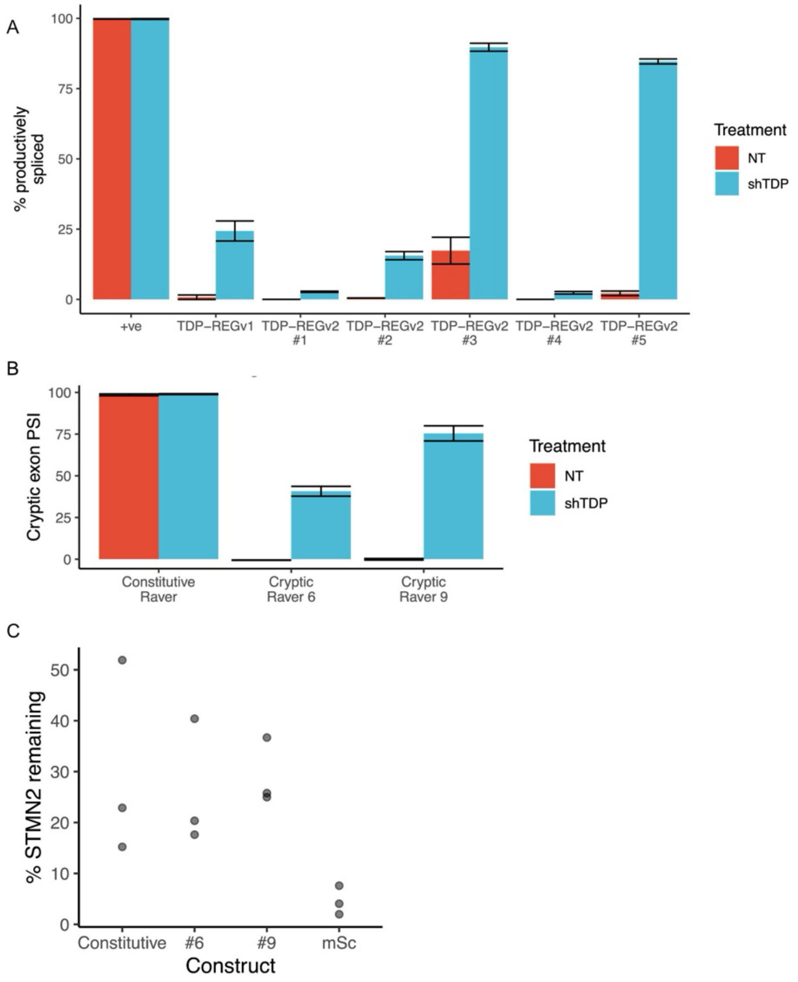

Fig. S8.

A: Nanopore sequencing of seven Luciferase constructs (one positive control, one TDP-REGv1, and five TDP-REGv2) with or without TDP-43 knockdown; error bars show standard deviation across replicates. **B:** Quantification of RT-PCRs detecting the internal cryptic exons present in TDP-REGv2 TDP-43/Raver1 constructs. In contrast with the data shown in Figure 4, in this figure the cells were stably expressing the vectors, and the TDP-43/Raver1 fusion protein was functional (i.e. without the 2FL mutation); error bars show standard deviation. **C:** Quantification of remaining STMN2 levels in three polyclonal SK-N-BE(2) lines of each construct (Constitutive TDP-43/Raver1, TDP-REGv2:TDP-43/Raver1 #6 and #9, and mScarlet) with doxycycline-induced TDP-43 knockdown; each value is normalized per-lane to tubulin, then normalized per-line to the untreated control.

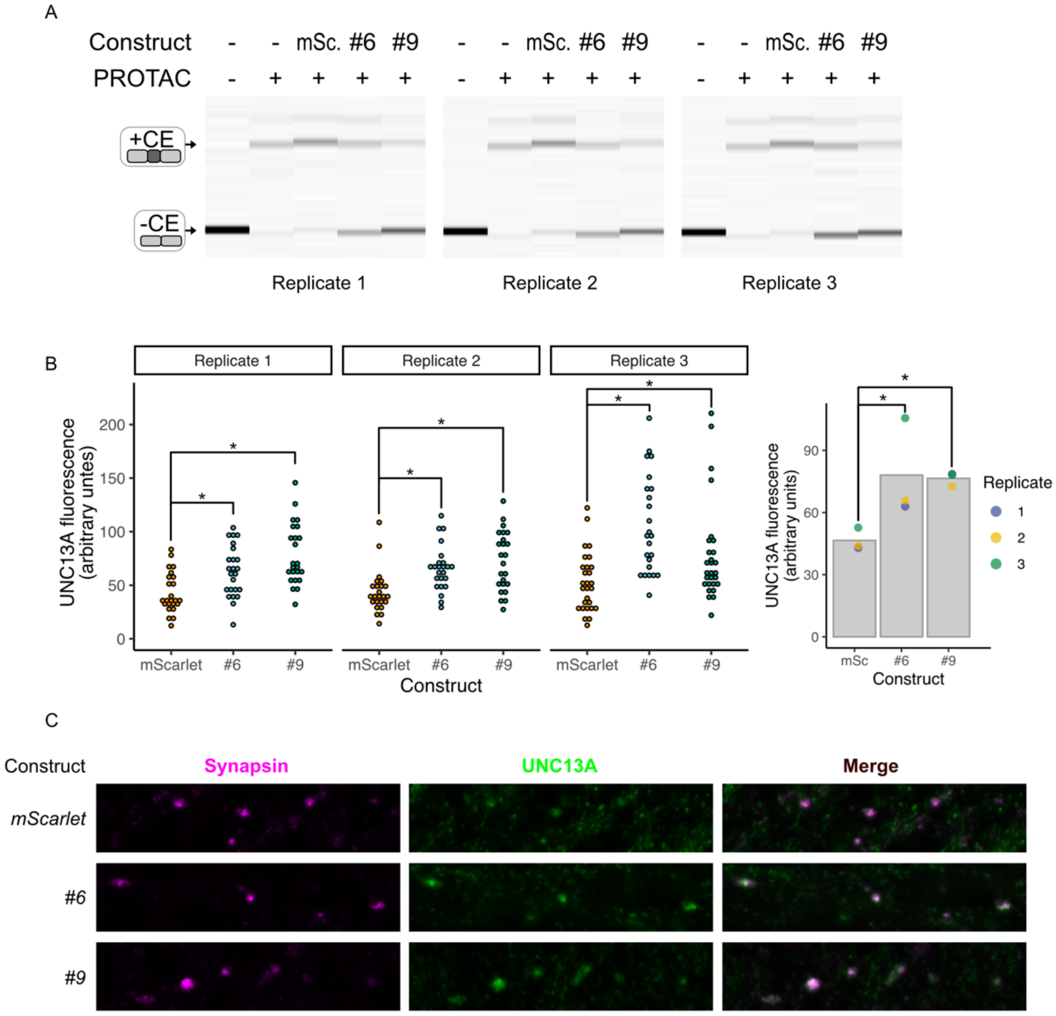

Fig. S9: A: Three replicates of RT-PCRs against the UNC13A CE for i3 cortical neurons, featuring Halo-tagged endogenous TDP-43 (thus enabling PROTAC-mediated degradation), with stably-integrated piggyBac vectors encoding either mScarlet or TDP-43/Raver1 (TDP-REGv2 TDP/Raver1 vectors #6 and #9); replicates are from separate wells. B: Quantification of UNC13A fluorescence in synapses formed by the piggyBac lines described in Part A. UNC13A staining is significantly lower for mScarlet lines than TDP-43/Raver1 #6 and #9 lines in all three replicates. Left: raw values from individual replicates (* = padj < 0.05; Dunn's test); Right: mean values from each replicate, bar graphs show mean of the three mean values (* = padj < 0.05, ratio t-test with Benjamini-Hochberg correction) C: Example fluorescence microscopy images used for the quantification in Part B, stained with antibodies against Synapsin (purple) or UNC13A (green).

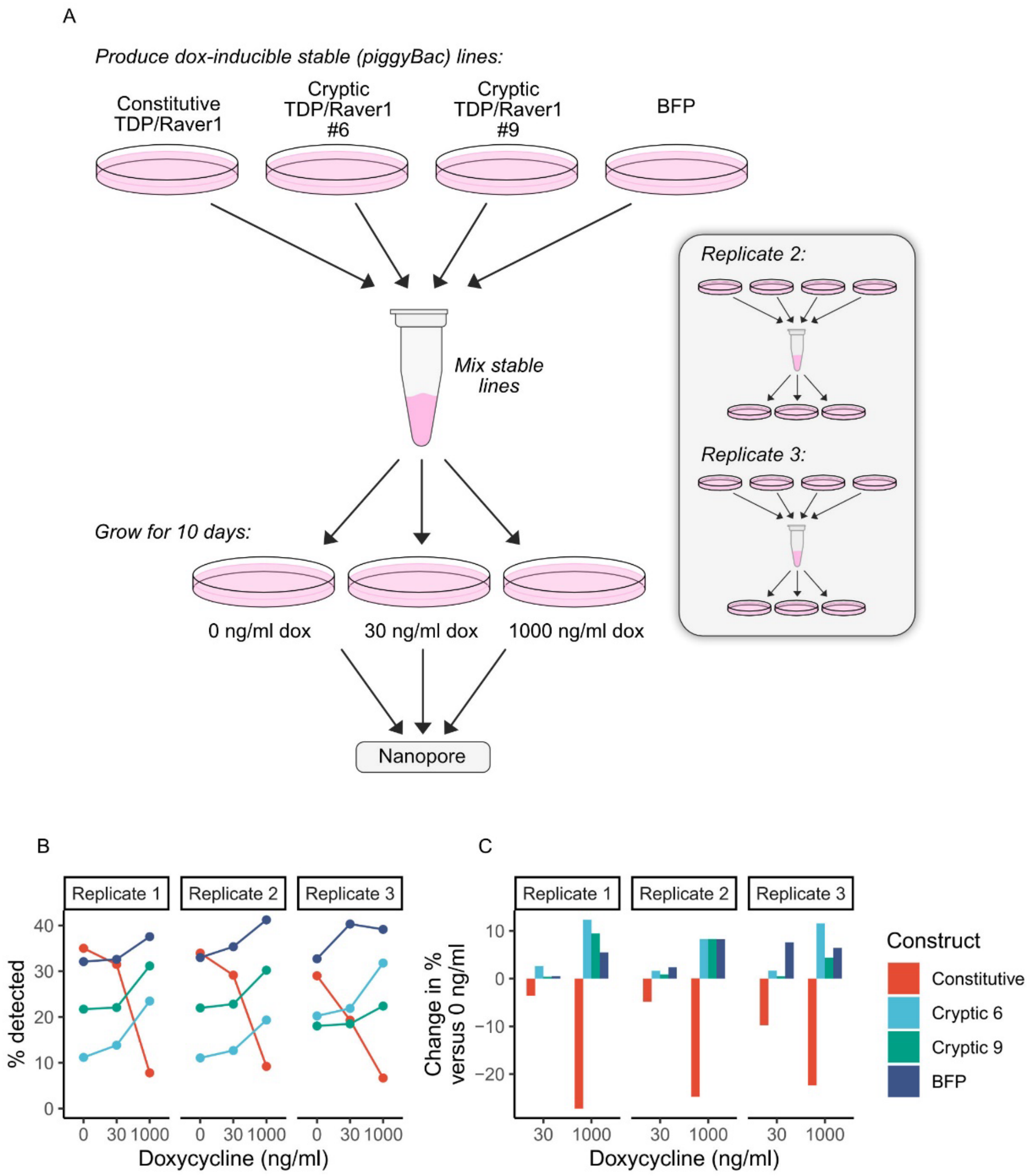

Fig. S10.

A: A schematic showing the experimental procedure for the growth competition assay. **B:** Quantification of Nanopore reads derived from each of the four constructs used to make stable piggyBac lines for each doxycycline concentration and each replicate. (Note that differences in % at 0 ng/ml doxycycline can be explained by PCR bias during Nanopore library preparation and unequal initial mixtures of the different lines; comparisons are only valid between doxycycline concentrations within the same replicate.) **C:** A second visualization of the same Nanopore data, where values for each construct are compared with their equivalent value when no doxycycline was added, for clarity.

Table S1.
Antibodies used in western blotting and microscopy experiments.

Target	Brand	Product code	Lot	Use
Anti-Mouse IgG1 (HRP)	Abcam	ab97240	GR3365481-1	Western blotting
Anti-Rabbit IgG H&L (HRP)	Abcam	ab6721	GR3242092-4	Western blotting
FLAG	Sigma	F3165	035K6196	Western blotting
α-Tubulin	Sigma	T5168	038M4813V	Western blotting
TDP-43	Proteintech	10782-2-AP	103682	Western blotting; immunofluorescence of neuroblastoma lines
Rabbit IgG (Alexa Fluor 647)	Abcam	ab150079	GR3444080-1	Immunofluorescence of neuroblastoma lines
TDP-43	Biolegend	808301	B305604	Immunostaining of tissue
RFP	Rockland	600-401-379	46317	Immunostaining of tissue
VAChT	Synaptic Systems	139105	4-26	Immunostaining of tissue
Rabbit IgG	Life Tech. Thermo	A-21207	2563838	Immunostaining of tissue
Rat IgG	Invitrogen	A21208	206333	Immunostaining of tissue
Guinea pig IgG	Invitrogen	A21450	2446026	Immunostaining of tissue
STMN2 polyclonal antibody	Proteintech	10586-1-AP	65379	Western blotting
Munc13-1	Synaptic Systems	126-104	1-7	Staining of synapses
Synapsin	Synaptic Systems	106-011	1-51	Staining of synapses

Table S2.
Details of animal experiments

See attached material

Table S3.
Primers used for RT-PCRs

Name	Sequence	Purpose
AARS1 forward	ACTTACTTTGGCGGGGATGA	RT-PCR of endogenous cryptic splicing
AARS1 reverse	AGGTTCCAGATCTCCAGCAC	RT-PCR of endogenous cryptic splicing
UNC13A forward	GTTCAAGAGGGAATCTGACG	RT-PCR of endogenous cryptic splicing
UNC13A reverse	GGGCACATATACTTGGAGGAG	RT-PCR of endogenous cryptic splicing
STMN2 forward	GCTCTCTCCGCTGCTGTAG	RT-PCR of endogenous cryptic splicing
STMN2 cryptic reverse	CTGTCTCTCTCTCTCGCACA	RT-PCR of endogenous cryptic splicing
STMN2 downstream reverse	CGAGGTTCCGGGTAAAAGCA	RT-PCR of endogenous cryptic splicing
check_tdp_splice_R_v2	ATTGCTGATGTGTACAGAGATGC	Analysing splicing of vectors encoding TDP-43/Raver1 fusion protein RT-PCR; amplifying TDP-43/Raver1 constructs in growth competition assay;
check_tdp_splice_F	GATTTGTCAGGTTCACTGAGTATGAG	Analysing splicing of vectors encoding TDP-43/Raver1 fusion protein RT-PCR; amplifying TDP-43/Raver1 constructs in growth competition assay
nRV_growthC_BFP_F	GGAGATCGATTCGGATG tcttcaagcagtccttccctg	Amplifying BFP construct in growth competition assay
nRV_growthC_BFP_R	GCCTTCCACTAGATTCC ACCCACTACCattaagcttgtgc	Amplifying BFP construct in growth competition assay
Cas9_splice_F	cgatctgctgaaaattatcaaggacaag	Check splicing of PE-Max vector via RT-PCR
Cas9_splice_R	tccaccaccttcactgtctg	Check splicing of PE-Max vector via RT-PCR

Table S4

See attached material
